# Inducible Pluripotent Stem Cells to Model and Treat Inherited Degenerative Diseases of the Outer Retina: 3D-Organoids Limitations and Bioengineering Solutions

**DOI:** 10.3390/cells10092489

**Published:** 2021-09-20

**Authors:** Massimiliano Andreazzoli, Ivana Barravecchia, Chiara De Cesari, Debora Angeloni, Gian Carlo Demontis

**Affiliations:** 1 Department of Biology, University of Pisa, 56127 Pisa, Italy; decesari.chiara@gmail.com; 2 Department of Pharmacy, University of Pisa, 56126 Pisa, Italy; barravecchia.ivana@gmail.com; 3 Institute of Life Sciences, Scuola Superiore Sant’Anna, 56124 Pisa, Italy; d.angeloni@santannapisa.it

**Keywords:** iPSC, retinal organoids, inherited retinal degenerations, photoreceptors, disease modeling, transplantation, subretinal space, aerobic glycolysis, bioengineering

## Abstract

Inherited retinal degenerations (IRD) affecting either photoreceptors or pigment epithelial cells cause progressive visual loss and severe disability, up to complete blindness. Retinal organoids (ROs) technologies opened up the development of human inducible pluripotent stem cells (hiPSC) for disease modeling and replacement therapies. However, hiPSC-derived ROs applications to IRD presently display limited maturation and functionality, with most photoreceptors lacking well-developed outer segments (OS) and light responsiveness comparable to their adult retinal counterparts. In this review, we address for the first time the microenvironment where OS mature, i.e., the subretinal space (SRS), and discuss SRS role in photoreceptors metabolic reprogramming required for OS generation. We also address bioengineering issues to improve culture systems proficiency to promote OS maturation in hiPSC-derived ROs. This issue is crucial, as satisfying the demanding metabolic needs of photoreceptors may unleash hiPSC-derived ROs full potential for disease modeling, drug development, and replacement therapies.

## 1. Introduction

**Figure 1:**
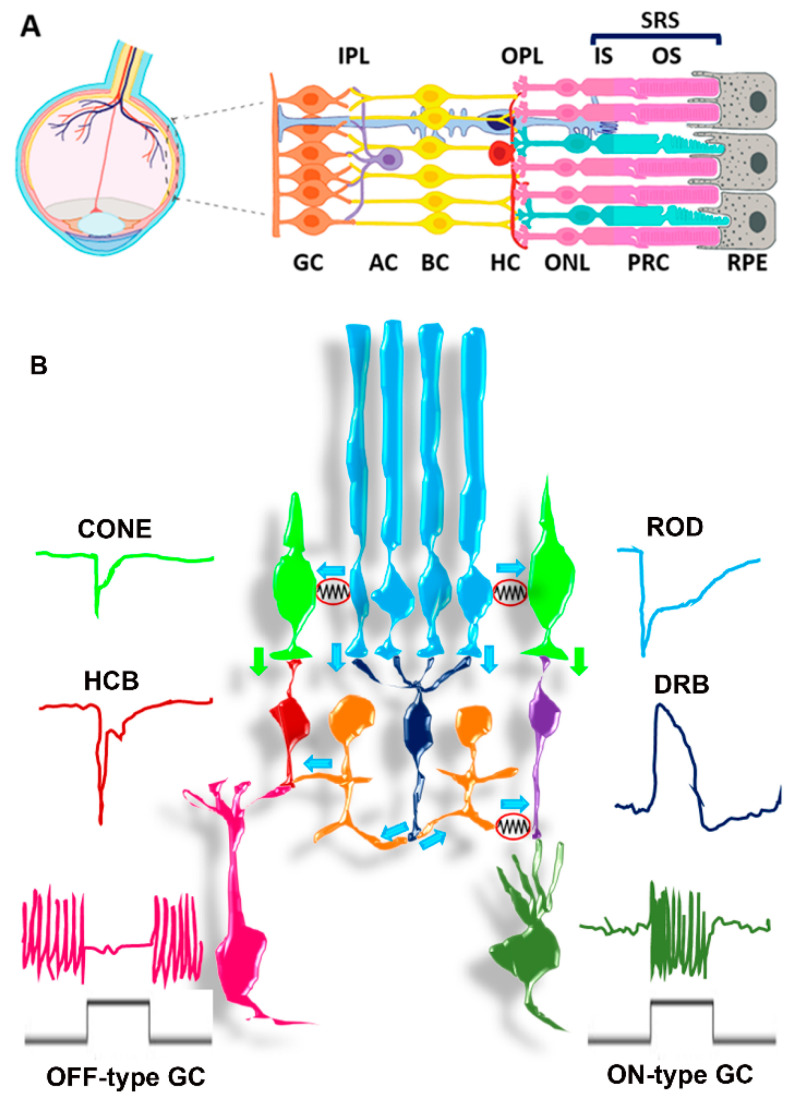
(**A**) In vivo cellular organization of the mature retina. The interactions occurring between distinct cell types and between photoreceptors and RPE are shown. GC: Ganglion Cells; AC: Amacrine Cells; BC: Bipolar Cells; HC: Horizontal Cells PRC: Photo Receptor Cells. IPL: Inner Plexiform Layer; OPL: Inner Outer Plexiform Layer. ONL: Outer Nuclear Layer; IS: Inner segment; OS: Outer Segment. The blue bar above IS and OS represents the SRS depth interposed between retinal pigment epithelial (RPE) cells and the end feet of Muller glial (MG) cells in the OR. The MG cell is shown in light blue and extends across the whole retina. (**B**) Parallel processing of PRC-generated signals by retinal networks. Cone cells (green) and hyperpolarizing response (on the left). Downward pointing green arrows: transfer of cone signals to hyperpolarizing CB (HCB) (red) and depolarizing CB (purple). Hyperpolarizing and depolarizing CB establish synaptic connection with OFF-type (pink) and ON-type (olive green) GC, respectively. Rod cells (cyan) and hyperpolarizing response to light on the right. Cyan arrows: transfer of rod-borne signals via primary and secondary rod pathways. Primary rod pathway: downward pointing cyan arrows indicate rod responses transfer to depolarizing rod BC (blue). Horizontal cyan arrows indicate the transfer of rod-borne signals to cone BC by AII AC (orange), via a chemical synapsis with HCB and an electrical synapsis (red circle containing a resistor symbol) with depolarizing CB. Secondary rod pathway: horizontal cyan arrows indicate the transfer of rod-borne signals to cones via electrical synapses.

The adult mammalian retina is a highly specialized tissue, localized in the posterior region of the eye, responsible for light sensing and pre-processing of the visual information that is readily delivered to the brain. As shown in Figure 1A, the retina has five major retinal cell types organized in three main cell layers separated by two synaptic layers. The visual experience starts in retinal photoreceptors (PRC) upon light stimuli conversion in electrical signals that feed into retinal circuitries for neural computation [1,2]. Specifically, PRC in the outer nuclear layer (ONL) transmit the neural signal to bipolar cells (BC) in the inner nuclear layer (INL) that relay this information to ganglion cells (GC), the only retinal cells whose axons exit the retina via the optic nerve. Amacrine and horizontal cells (AC and HC) in the INL interconnect with either PRC, BC, or GC to integrate PRC-born signals, letting the visual system operate over a range of environmental light intensities spanning about 14 log units as parallel networks [3] extracting relevant features from the visual scene [4,5]. Figure 1B provides an example of how retinal circuitries let the visual system operate over a large range of light intensities via the parallel processing of rod and cone signals (reviewed in [6]). As shown in Figure 1B, cones hyperpolarize in response to light and connect with BC that either hyperpolarize (hyperpolarizing cone BC—red) or depolarize (depolarizing cone BC—purple) in response to light. Hyperpolarizing BC cells connect to OFF-type GC that reduce their firing rate in response to light. In turn, depolarizing cone BC connect to ON-type GC that increase their firing rate in response to light. Through these, ON and OFF GC cone-borne signals are relayed to higher visual centers enabling vision in day-light. However, cones do not have enough sensitivity to operate in dim light levels, preventing common tasks such as driving a car at night. Rods (cyan) hyperpolarize in response to light and have enough sensitivity to operate in dim light and connect with rod BC (blue) that respond to light with a depolarization. Rod BC do not establish synaptic contact with GC and rod-born signals travel through a specialized AC type (bistratified or AII AC-orange) to reach cone bipolar cells (Figure 1B). In addition to this primary rod pathway, a second pathway directly connects rod to cones (Figure 1B).

In the case of Inherited Retinal Degenerations (IRD) affecting photoreceptors and the retinal pigment epithelium (RPE), the progressive visual loss eventually leads to severe blindness with a prevalence of about 10% [7].

The RetNet database (https://sph.uth.edu/RETNET/sum-dis.htm, accessed on 15 February 2021) reports 307 genetic loci associated with visual impairment, and most genetic loci have been linked to a single gene. Table 1 reports data extrapolated from the RetNet database, after excluding syndromic cases (i.e., involving disorders in other organs), non-progressive visual disturbances, and visual loss involving damage to extraretinal eye structures. The high numbers of involved loci (130) and identified genes (114) responsible for Retinitis Pigmentosa (RP), Leber Congenital amaurosis (LCA), Cone or Cone-Rod dystrophy (CORD), and Macular Degeneration (MD) indicate the genetic heterogeneity of IRD.

Several genes in the RetNet database involved in early- or late-onset IRD play a role in either PRC development or function. Those mentioned in this review are listed in Table 2 below.

Despite the impressive progress in identifying the genetic basis of IRD, translation of basic science into effective treatments validated by clinical studies largely remains a work in progress. This review is organized in four main sections. In the first one, we will recapitulate the main issues in drug development and cell replacement strategies to treat or cure IRD in order to restore vision (reviewed in [8]). In the same section, we will focus on the potential of inducible pluripotent stem cells (iPS) and iPS-derived three-dimensional (3D) retinal organoids (ROs) to promote drug development [9,10,11,12,13] and substitutive approaches to IRD by improving disease modeling [9,10,11,12,14,15,16,17,18,19,20,21,22,23,24] and cell replacement therapies [12,25,26,27].

In Section 2, we will cover the significant improvements in hiPSC-derived ROs generation via culture protocols recapitulating retinal embryogenesis and leading to three-dimensional structures with ordered retinal cells layering. Moreover, we will also address the unsolved issue of photoreceptors maturation in hiPS-derived ROs and critically review recent data on the functional properties of hiPS-derived ROs.

In Section 3, we will review evidence from the mammalian retina pointing to the critical role of the subretinal space (SRS) microenvironment to support PRC metabolism critical for their maturation. In Section 4, we will cover the bioengineering approaches to reproduce the SRS and to improve donor photoreceptor transplantation to the SRS.

### 1.1. Disease Modeling by hiPSC-Derived ROs and Drug Development for Retinal IRD

Drug-based approaches may overcome the problems generated by the large number of variants each one affecting a relatively small number of patients worldwide by targeting apoptosis. Apoptosis, indeed was originally proposed as the final common pathway in rod degenerations induced by defects in different biochemical mechanisms [28,29]. However, this notion was challenged by the discovery that multiple apoptotic pathways [30], microglia phagocytosis [31], necroptosis [32], autophagia, and their combinations [33] contribute to rods demise (recently reviewed in [34,35].

An additional challenge to the notion of a single apoptosis-based mechanism in rod loss is the evidence from animal models that distinct mutations of a single gene may cause rod cell death via either apoptosis or necroptosis. The P23H variant of *Rho* (coding for the light-sensitive protein rhodopsin) causes rod demise via necroptosis and cone loss via inflammasome activation [32], with necroptosis occurring independently from caspases and AIF activation [33]. In turn, the *Rho* S334ter variant causes rod death via caspases activation and induces cones loss via necroptosis [32].

Inflammation in RP patients [36,37] may cause microglia activation, which precedes apoptosis in animal models of RP [36,38,39]. Indeed, inhibition of microglial cells activation by minocycline attenuates rd10 rod degeneration [40], and activation of microglia-expressed C-X3-C Motif Chemokine receptor 1 (CX3CR1) by its ligand (CX3CL1, fractalkine) [41] reduced inflammation, microglia infiltration of ONL, and altogether rod demise [40,42].

In summary, these data indicating inflammation and microglia activation precede apoptosis in some RP models make somewhat unrealistic the idea of developing a single drug to target a common molecular pathway to prevent PRC loss. In addition, considering the evidence on multiple mechanisms underlying PRC demise in IRD animal models, the development of human disease models through hiPSC-derived ROs (reviewed in [8,16,17,18]) may prove strategic to devise effective treatments as well as the time window for their effectiveness (for an updated and detailed review on the use of human ROs for disease modeling, see [43]).

In recent years, hiPSC-derived ROs and optic cups have been used to evaluate the basis of diseases associated with early-onset IRD. These mechanisms include the interplay between MITF and VSX2 and their respective regulators Wnt/βCatenin and FGFs, in the cell fate decision concerning neural retina versus RPE, during optic cup formation [24,44,45,46]. Moreover, several retinal pathologies have already been modeled in human organoids generated from patient-derived cells. In this way, it has been possible to study the disease-associated mechanisms caused by mutations in genes encoding for Retinitis Pigmentosa GTPase regulator (RPGR), receptor expression enhancing protein 6 (REEP6) and X-linked Retinitis Pigmentosa 2 (RP2) [47,48,49,50] as well as by an intronic mutation in the *CEP290* gene encoding for a centrosome-cilia protein, responsible for LCA [51]. Furthermore, phenotypes of mutations in the CRB1 gene, leading to disruption of the OLM [52] (see Section 3.1) and in the RS1 gene, causing X-linked juvenile retinoschisis [53], have also been recapitulated in organoids derived from specific patients. Additional models include adRP [54], non-syndromic RP [44,49,55,56,57], and LCA [51].

Some of these studies also successfully used hiPSC-derived ROs to evaluate the efficacy of different strategies aimed at rescuing or compensating the activity of the mutated gene. Strategies include CRISPR-Cas9-mediated correction of the mutated gene [58,59], antisense morpholino to block aberrant splicing [51], read-through promoting drugs to treat frameshift mutations [56,60], and treatment with neuroprotective factors [53,61].

However, these disease models relate to early-onset IRD, and a meaningful comparison to animal models or human samples may require ROs from healthy subjects or isogenic controls with well-developed photoreceptors. Although this point appears especially critical for late-onset IRD, even for a model of early-onset IRD, the lack of well-developed ROs may blur important long-term difference between controls and patients and affect the development of effective therapies.

### 1.2. hiPSC-Derived ROs and Replacement Therapy for Retinal IRD

Replacement therapy aims to cure IRD by replacing dead host cells with transplanted donor cells. The cell replacement approach is conceptually attractive and straightforward, overcoming the main limitations of gene therapy and drug-based approaches: it represents a variant-independent approach that may work when host cells have already degenerated, i.e., when drug therapy could not work. However, replacing dead cells in the degenerated retina proved more difficult than anticipated (for recent detailed reviews on replacement therapies for retinal degenerations, see [62,63]).

Clinical trials in RP patients using retinal sheets of the human fetal retina with attached RPE provided evidence for safety and lack of rejection, despite human leukocyte antigen (HLA) mismatch. Efficacy appeared limited [64], lasting up to 5 years post transplants in some RP patients [65] (reviewed in [66]). Importantly, transplantation of hESC-derived ROs slices lacking RPE cells to the SRS of nude rat model of rapid (Rho S334ter-3 rats with a truncation in the C-terminus causing endoplasmic reticulum stress) [67] and late IRD (RCS rats with faulty OSs phagocytosis by the RPE) [68] led to visual improvements despite human rods and cones organizing in rosettes [67,68]. Consistent with poor transplantation outcomes in the same rat model when human fetal strips generate rosettes [69], the visual improvement conferred by transplanted human ROs strips was transient [68], suggesting their reduced viability upon transplantation to the SRS.

These clinical and preclinical studies using fetal and hESC-derived ROs slices did not address the issue of the optimal developmental timing for transplantation. Instead, this was addressed in preclinical models of retinal transplantation using post-mitotic rod precursors isolated from the retina of transgenic Nrl:GFP mice [70], which express the green fluorescent protein (GFP) under the control of the rod-specific Neural Retina Leucine Zipper (NRL) transcription factor [71].

These studies indicated a higher success rate for rod precursors from retinas of post-natal (PN) PN4-PN7 mice donors [72] compared to rod precursors isolated from adult mice [73,74]. Although the host retinal environment emerged as an additional variable affecting transplantation outcome [75] (reviewed by [76]), transplanted photoreceptor precursors improved vision in both non-degenerative (night-blindness) [77] and end-stage degenerated retina [78].

Several pre-clinical studies using iPSCs-derived donor cells (reviewed in [79,80]) have also addressed open issues about cell replacement approaches to IRD. Evidence that rod and cone precursors generated from hiPSC cells may develop IS and OS upon transplantation to the SRS of recipients [81,82,83] represented a significant step toward translating preclinical cell replacement approaches into clinical applications. However, the finding that, upon transplantation of hESC-derived retinal cells, *Crx*^−/−^ recipient mice became light responsive, despite grafted cells failing to develop an OS [84], represents a puzzling observation bearing relevance to the functional evaluation of hiPSC-derived ROs. Specifically, in response to light stimuli, *Crx*^−/−^ mice recipients generate an ERG b-wave, indicating the operation of the synapse between PRC and ON-bipolar cells, a finding consistent with the expression of the presynaptic marker synaptophysin by grafted cells. Overall, these data suggest some grafted cells establish functional synapses with postsynaptic ON-bipolar cells. The puzzling finding is that PRC lack OS, and accordingly, the ERG lack a discernible a-wave, the component ascribed to the suppression of the dark current circulating between the OS and the IS [85,86,87] (reviewed in [88]). Importantly, light-responses in the absence of well-developed OS have also been reported in 6–7 months rds mice [89], and b-wave amplitude in P23H rats poorly correlates with ONL thinning [90]. In mice, immature rods start expressing *Rho* before OS formation, and rhodopsin immunostaining reveals labeled cell bodies. In case OS fail to develop, opsin may accumulate in the cells’ body and becomes photosensitive upon linking RPE-generated 11-cis retinal. In these conditions, PRC may become light responsive despite the lack of OS and ERG a-wave.

An interesting finding of this early work was the observation that upon transplantation to the SRS, hESC-derived retinal cells may integrate into the retina of wt mice and human PRC developed OS with the morphologies of wt mice, a striking difference with the finding in *Crx*^−/−^ mice. It is now clear that in some IRD models, material transfer between grafted photoreceptor precursors and host rods [91,92,93] and cones [94,95,96] represents the primary mechanism contributing to host cell rescue [95] by grafted cells and visual improvement in some models of IRD.

An additional mechanism, distinct from material transfer from grafted rod precursors, is the one reported for late retinal progenitors, isolated as c-KIT+/SSEA4− cells from human ESC-generated ROs. These progenitors may contribute to cell rescue via both cell replacement and by material transfer [97], as well as by suppressing microglia activation along with inflammatory cytokines production. Overall, these data indicate cell rescue rather than cell replacement may represent the main mechanism underlying preserved function in transplantation, depending on the developmental age of grafted cells, indicating the importance of selecting cells of precise developmental stages.

It should also be considered that human rod precursors, which develop late in the second trimester of gestation [98,99], may not be available for transplantation. Moreover, photoreceptor precursors isolation from ROs requires transgene-independent sorting strategies. Based on preclinical studies showing the successful isolation of mouse rod precursors using the cell surface marker CD73 [100], additional work evaluated several CD combinations [101,102]. However, the sorting selection based on several CD markers gave similar results to cells selected on CD73/CD24 combination, with engrafted cells barely reaching 1% of donor cells [102]. Importantly, similar sorting strategies have been found effective in isolating human rod precursors from hiPSC-derived ROs, and sorted cells did survive in the SRS following transplantation [103].

The similar engraftment yields with different and progressively more selective sorting criteria challenge the notion of an optimal maturation stage for engrafting and point to a role for stage-independent mechanisms. In line with this notion, the successful engraftment of donor cone precursors generated from hiPSC-derived ROs upon transplantation in an end-stage model of retinal degeneration has recently been achieved [104], with donor human cone precursors becoming light-responsive and establishing synaptic connection with postsynaptic cells has recently been achieved [104]. An important difference with previous transplantation work, where cone precursors survived in the SRS but failed to mature OSs, was the increased donor cell number (5 × 10^5^ cells/1.5 µL) transferred to the SRS [104] compared to previous studies (1 × 10^5^–2.5 × 10^5^ cells/1–2 µL, see Table 1 in [62]). These findings hint to a role for cell–cell contacts or short-range diffusing molecules in affecting maturation of grafted cells in the SRS.

In summary, multiple mechanisms may contribute to visual improvement upon transplantation of PRC precursors isolated from hiPSC-derived ROs, ranging from cell rescue to cell engraftments. PRC precursor maturation stages play an important role, but the number of grafted cells also appears to have an additional effect in promoting integration.

### 1.3. IRD Treatments: What Role for hiPSC-Derived ROs in Addressing Current Issues?

The possibility of deriving hiPSCs from readily available sources such as skin or blood offers the opportunity to generate HLA-matched ROs with specific genotypes, allowing personalized treatments through the development of in vitro 3D models of retinal diseases [105,106,107,108]. Indeed, as already discussed in Section 1.1 and Section 1.2, studies performed on patient-derived ROs have cast light on the molecular mechanism underlying human retinal development, otherwise very difficult to investigate. However, studies with hiPSC-derived ROs and optic cups have so far mostly focused on early events in retinal development, such as defects in VSX2 [44,46] and MITF [45] genes or on the role of retina-specific transcription factor isoforms (PAX6D) [109], the epigenetic impact of variants associated with RP [54], or diffusible factors such as FGF9 [110]. Analysis of the impact of defects affecting genes expressed later during development, such as those involved in protein trafficking at the connecting cilium between IS and OS have recently been attempted [50,55,60,111] (reviewed by [17]). However, few human disease models requiring an advanced degree of PRC functional maturation are available, such as those involving phosphodiesterase or cGMP-gated channels defects. Animal models of *PDE6B* defects, such as the Rd1 mouse, have been found to involve multiple mechanisms [112,113,114,115] (reviewed in [35]), with apoptosis lagging behind microglia activation [38,39,42]. hiPSC-derived ROs may provide knowledge of disease progression in humans rather than animal models, a key step for testing novel drug combinations tuned to specific cascade events [11]. A recent study generated hiPSC-derived ROs from patients bearing *PDE6B* mutations, and found evidence for developmental defects at day 230 in culture, i.e., w32–33, when photoreceptors mature in the human retina. However, considering that cGMP elevation caused by reduced PDE6B activity may have pleiotropic effects [112,113,114,115] (reviewed in [35]), the extent to which these ROs will recapitulate the human phenotype critically depends on the level of morphological and functional maturation of their PRC.

As the number of retinopathies modeled in organoids is rapidly increasing, drug discovery is another field in which human ROs can give a crucial contribution, providing information complementary to 2D culture systems and animal models. However, the application of human ROs in drug screening still presents several drawbacks, mainly the heterogeneity of the organoids and extended developmental time, which spans from several months to over one year, similarly to human embryo developmental timeline. Laborious protocols not easy to automate, expensive reagents, and scarcity of quantitative assays suitable for organoids represent additional challenges (reviewed in [13,116]).

The opportunity to use ROs as a renewable and scalable source of photoreceptors for transplantation in patients affected by degenerative retinopathies has been explored by transplanting organoid-derived retinal sheets [117,118] as well as purified rod precursors [119,120] in mice models of retinal degenerations.

Optimizing donor cell engraftment [121] may require an improved knowledge over the intrinsic and extrinsic factors controlling PRC maturation.

In this view, it is strategic to address the current limitations of three-dimensional (3D) ROs to improve their usefulness as disease models for tailoring and testing new drug combinations tuned to specific gene variants at a specific time of disease course (reviewed in [12]), as well as their use as donor cells in regenerative approaches.

In the next sections, we will review how what we learned from retinal development could be applied to hiPSC to generate 3D ROs and outline the problems that limit ROs application to disease modeling and transplantation studies. We will then move to recent technological advances that provide new perspectives on improved use of hiPSC and 3D ROs.

## 2. The Path from Retinal Development to ROs

The mammalian retina is a highly specialized tissue, whose complex functional structure is progressively shaped and organized due to well-orchestrated events occurring during embryonic development of the nervous system. The territory fated to give rise to the retina, the eye field, is first defined in the neural plate anterior-most region. Following neurulation, two lateral regions of the diencephalon evaginate to generate the optic vesicles, which in turn indent to form the optic cups, two-layered structures with the inner layers (walls) giving rise to the retina surrounded by external layers fated to become the RPE. The pool of multipotent retinal progenitor cells (RPCs) expands by symmetric cell divisions, and through asymmetric cell divisions, they eventually give rise to all the five principal retinal neurons (see Figure 1). The retinal cell types are generated according to an evolutionary conserved temporal order, in partially overlapping waves of cell specification. The cell bodies of each differentiated retinal cell type are localized in specific positions so that the mature retina organization is constituted by three cell layers connected by two plexiform layers, as shown in Figure 1A.

The great majority of our knowledge about the cellular and molecular mechanisms governing mammalian retina development and differentiation arises from studies performed in the mouse. Nevertheless, these studies have been preceded by functional analysis carried out in more accessible lower vertebrate model systems, particularly the frog *Xenopus laevis*, which led to discovering evolutionary conserved key regulators of the different phases of retina formation [122]. Altogether, this work unraveled the retinal development control exerted by the coordinated actions of intrinsic regulators of genetic programs, mainly including transcription factors and chromatin regulators, as well as extrinsic signaling factors (reviewed in [123]). An interesting example of the role of secreted factors is the conversion by the secreted protein Noggin of *Xenopus laevis* animal cap pluripotent stem cells into retinal precursors, which upon transplantation develop into eyes with functional photoreceptors [124,125].

### 2.1. Derivation of 3D ROs from iPSC

The information gathered from lower vertebrate model systems has been crucial to defining protocols driving mouse and human pluripotent stem cells toward a retinal fate.

In particular, the knowledge that inhibition of BMP and Wnt pathways lead to forebrain induction, while IGF1 instructs neural progenitors to adopt a retinal fate, proved fundamental to develop retinal differentiation protocols for 2D adherent cultures [126]. However, these cultures did not recapitulate the complex cell–cell interactions and exposure to gradients of diffusible factors that naturally occur in the retina.

A pivotal breakthrough was represented by the pioneering work of Yoshiki Sasai, which led to the generation of the first self-organizing mouse 3D optic cups in vitro. The original protocol used 3D aggregates of Pluripotent Stem cells (either ESC or iPSC), called embryonic bodies (EBs), which are maintained in serum-free, floating conditions in the presence of low concentrations of growth factors and Matrigel as a source of extracellular matrix components [127]. This protocol allows the spontaneous formation of optic vesicles and, following invagination, to optic cup-like structures, which are then excised from the aggregate and develop individually to an inner multilayered neural retina and an external RPE. Furthermore, dorsoventral and apical-basal polarities are established within the self-formed retinal neuroepithelium, and retinal progenitors display the interkinetic nuclear migration typically observed in vivo. These morphogenetic events occur in vitro in the absence of cues or forces from external structures, such as the surface ectoderm or the lens, indicating self-driven morphogenesis mechanisms.

The pursuit of potential future therapies for retinal neurodegenerations made apparent the necessity to establish human ROs. This goal was initially achieved by the Sasai’s group that, taking advantage of knowledge gained in earlier 2D studies, adapted their original mouse protocol to human ES cells [128]. Main features of this new protocol were the presence in the medium of 10% FBS and the addition of a Wnt inhibitor, as anteriorizing factor, a Hedgehog agonist, to promote retinal differentiation, and a ROCK inhibitor to prevent dissociation-induced apoptosis.

Despite the invaluable importance of this groundbreaking discovery, several groups found that the original Sasai protocol displayed a remarkable heterogeneity, reflected in a variable efficiency in generating invaginated optic cups and appropriate cell layering. To improve these aspects, as well as to increase the scale production of ROs, distinct modifications of the original protocol were adopted by different laboratories. Although these improvements are continuously evolving, leading to a large variety of published procedures, most protocols stem from two major approaches: embryoid body formation [82,128,129] and a mixture of 2D and 3D protocols [14,130,131,132]. Comparisons between these different types of protocols have been recently reviewed [11,24,116], and it is interesting to note that, regardless of the presence or absence of growth factors and steps including cultures in adherent or in suspension conditions, virtually all protocols include a neural induction step and the subsequent isolation of neuroepithelia, followed by experimental conditions that promote early and late phases of retinal cell maturation.

Overall, a comprehensive analysis of the different studies highlights the protocol and indicates also potential factors that could overcome the problems.

An important issue concerns the variable efficiency in generating differentiated retinal organoids displaying an appropriate layering. This heterogeneity has been shown to depend on different epigenetic states of the source cell lines, resulting in different levels of endogenous signaling activities and expression of retinal lineage markers [119,120,133,134,135,136]. As a consequence, the same protocol may show different efficiency in distinct cell lines, depending on whether the integration between the added factors and the endogenous levels of key retinal regulators in the starting population of iPSCs reaches the optimal concentration.

Generation of functional photoreceptors is another crucial topic in ROs protocols, which has been addressed by regulating specific signaling and differentiation pathways at different in vitro developmental times. In particular, maturation of photoreceptors can be promoted by retinoic acid and by modulating oxygen concentration [82,117,119,137]. Furthermore, inhibition of Notch signaling enhances either cone or rod generation, depending on the developmental temporal window of treatment [138], whereas opsin-specific 9-cis-retinal promotes rod generation [139]. Another factor recently shown to be influential for the generation of retinal organoids is the starting cell number, which should be in the optimal range between 6000 and 8000 cells. Finally, several studies investigated the role of extracellular matrix in the generation of ROs. The distribution of key components of retinal extracellular matrix (ECM) was found to be very similar in developing retina and ROs, while functional data indicated that CD44 and the photoreceptor-specific ECM IMPG1 are necessary for the development of photoreceptors in human ROs [140]. To better mimic the natural functions of ECM, which provides structural support and a source of biochemical and physical cues necessary for cell survival and differentiation, tissue decellularization protocols have been established. In particular, cell culture media supplemented with decellularized ECM from neural retina and retinal pigment epithelium have been shown to significantly increase photoreceptor differentiation, synaptogenesis and light responsiveness in human iPSC-derived ROs [141]. In a different approach, which uses biomaterial scaffold as substitute for ECM, alginate hydrogels, but not hyaluronic acid-based hydrogels, were shown to enhance the generation of retinal cells in ROs [142].

### 2.2. Morphological and Transcriptomic Limitations of iPSC-Derived ROs

The potential inherent in ROs is unprecedented, especially in the field of human retina research. However, despite the significant progress in the RO generation, molecular characterization, and applications, some critical issues persist and need to be overcome to unleash the full potential of these 3D in vitro models.

A major problem concerns the impaired differentiation of distinct cell types. Retinal GC are among the most affected, as they are initially generated but then progressively lost, possibly due to the lack of synaptic interactions with brain targets. In long-term culture, interneurons of the INL are also lost, probably as a consequence of trophic deprivation caused by loss of a synaptic partner, resulting in a disrupted inner layer lamination.

An issue of particular relevance for the development of human models of outer IRD concerns the functionality of photoreceptors, as these are among the cell types primarily affected in degenerative retinopathies.

**Figure 2:**
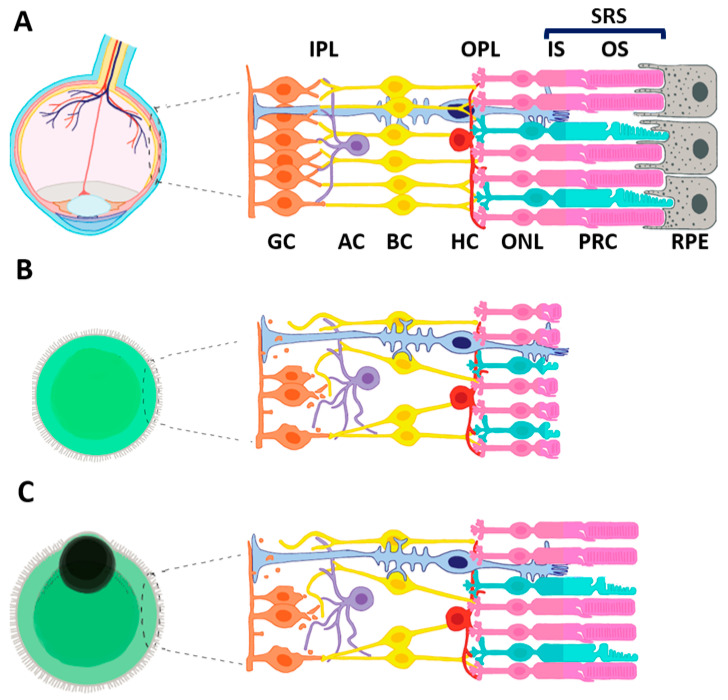
(**A**) In vivo cellular organization of the mature retina. The interactions occurring between distinct cell types and between photoreceptors and RPE are shown. See Figure 1A legend. (**B**) ROs lacking RPE display photoreceptors with severely reduced OS and progressive loss of RGCs and interneurons. (**C**) ROs with a patch of RPE localized on one side develop photoreceptors with distinct OSs, although shorter than those observed in the retina. Progressive loss of RGCs and interneurons is also observed.

As shown in Figure 2A, mature, healthy PRC are distinctively characterized by their OS, a highly specialized light-sensitive ciliary organelle where phototransduction takes place. OS are dynamic structures undergoing continuous renewal. New disc membranes are added at the base, and old discs shed from the distal portion phagocyted by the overlaying RPE cells [143].

Among the genes associated with RP in the RetNet database, several do code for proteins involved in phototransduction (*CNGA1*, *CNGB1*, *PDE6A*, *PDE6B*, *PDE6G*, *RHO*, *SAG*, *ABCA4*) and for cilium-associated proteins involved in OS morphogenesis and maintenance (*ARL6*, *ARL2BP*, *C2ORF71*, *RP1*, and *TTC8*). Therefore, it is essential for transplantation purposes and disease modeling to generate organoids with fully functional photoreceptors.

However, ROs generated with the original protocols featured photoreceptors that lacked discernible OS [127] (Figure 2B), indicating that the conditions used for differentiation in vitro differ from those present in the developmental niche of these cells [129]. More recent improvement of protocols resulted in generating photoreceptors carrying cilia and nascent OS-like structures containing membranous discs (Figure 2C), albeit disorganized [82,132,136,144] (reviewed in [17]). A crucial role in photoreceptor maturation and rhodopsin expression is played by retinoic acid, although cones and rods appear to have different requirements for this molecule, and the time window of organoid treatment must be finely modulated [132,145,146].

In the mouse retina, a transcriptomic analysis indicated that a switch takes place at PN6 when genes controlling neuronal generation are downregulated and those involved in phototransduction become upregulated [147]. Interestingly, subsequent analysis highlighted that, although gene expression of mouse iPSC-derived ROs during cell lineage specification largely overlaps in vivo retinogenesis, significant discrepancies exist at times ranging from PN6 to PN10 [148]. In particular, the transcriptome shift, involving the coordinated regulation of genes required for photoreceptor OS, synapses, and OPL formation, seemingly does not occur in mouse ROs, displaying dysregulation of multiple transcription factors controlling progenitor proliferation and cell differentiation timing [149,150,151].

In addition to the intrinsic factors controlling retinal development and postnatal maturation, the misplaced RPE (Figure 2C), failing to establish a close and functional connection with ROs, may cause a lack of soluble factors involved in photoreceptor survival, such as the polyunsaturated fatty acid docosahexaenoic acid (DHA) and FGF1. This lack of diffusible factors is a possible contributing cause for incompletely developed OS [148]. However, the limited improvements in photoreceptor maturation observed after DHA and FGF1 treatment of ROs indicate the necessity to identify additional signaling factors provided by RPE. RPE cells may nevertheless be important for ROs development even without either close contact with photoreceptors OS or the release of DHA or FGF1, as it was reported that hiPSC-derived ROs, with attached patches of RPE at their periphery, attain an expression profile similar to the adult human retina by 30–38 weeks in culture [136].

### 2.3. Functional Limitations of hiPSC-Derived ROs

Although the transcriptomic profile of ROs provides important cues on the generation of specific cell types and their relative ratios, the extent to which these molecular data provide insights into function remains unclear. As several genes involved in IRD code for proteins implicated in phototransduction, modeling and drug testing for IRD arising during late developmental stages require ROs with advanced functionality. It should be noted that the transcriptome of human ROs after 38 w in cultures approaches that of the adult retina, but ROs do not generate long OS [136]. This observation may indicate the functional maturation of human ROs lag behind the acquisition of mature molecular signature and may require additional extrinsic factors not available in their present configuration. It is therefore important to evaluate ROs from a functional perspective, as transcriptomic data may provide insights on necessary, but not sufficient, factors for their maturation.

The first functional analysis of hiPSC-derived ROs was carried out in ROs generated through a modification of the mixed 2D-3D protocol [14,152,153]. After 27–28 weeks (w27–28) in culture, these ROs generate photoreceptor precursors with cilia, IS, and some of them (on average 2 PRC every 150 µm of ONL length) also rudimentary OS with few stacked disks [132]. These morphological features closely resemble those of human photoreceptor precursors in the last month of the 2nd trimester (w21) of pregnancy [98,99]. At w25, photoreceptor precursors in ROs also express several components involved in phototransduction, such as the α (CNGA1) and β (CNGB1) subunits of the rod cyclic-nucleotide-gated-channel (CNG), the retinal guanylate cyclase 1 (RETGC1) required to generate the second messenger activating the CNG channel, as well as the α-subunit of rod transducin (GNAT_1α_) required for activating the rod cGMP-phosphodiesterase (PDE6), composed of α- and β-subunits, to close CNG channels [132]. Perforated patch-clamp recordings (to preserve the intracellular environment) in 13 w27–28 PRC from human ROs indicated a large inward dark current suggestive of open CNG channels in 3 PRC. Stimulation with a bright light suppressed a fraction of the large inward dark current in 2 out of 3 cells, suggesting that a fraction of PRC is light-responsive [132].

Although these results indicate that PRC in hiPSC-derived ROs are light-responsive, it is also important to note some limitations. First, the PRC failed to respond to a second stimulus, likely as a consequence of the lack of nearby RPE cells necessary to regenerate the bleached pigment on a time scale of minutes after a bright stimulus. Response amplitudes represent an additional issue, as the values recorded from photoreceptor precursors in ROs (range 20–30 pA), are similar to those recorded with the suction pipette technique [154] from adult primates PRC, whose OS length exceeds 20 µm [155,156]. Considering that response amplitudes are proportional to OS length [154], after accounting for the fraction of the dark current collected by the suction pipette, it is unclear whether responses recorded from photoreceptor precursors in ROs result from the closure by the transduction cascade of CNG channels open at their short OS. Finally, bright light appears to close a fraction only of the dark current in responding cells, although the long time-course of response recovery at 35 °C rather suggests all CNG channels had been closed by the bright light. These functional data are hard to reconcile with the properties of adult primate PRC, and perhaps reflect the functional immaturity of photoreceptor precursors in ROs.

The notion of immature photoreceptor precursors in hiPSC-derived ROs is consistent with the analysis of voltage- and cGMP-gated currents carried out at different developmental time of ROs generated with the same mixed 2D–3D protocol used to generate ROs with functional PRC [132]. Each cell type in the body expresses a unique combination of ion channels that represent its electrophysiological fingerprint and provide a key to its identification. Both rods and cones in the adult retina express a similar combination of genes coding for voltage-gated currents that include: *HCN1*, *KCNB1*, *KCNV2*, *CACNA1F*, *CACNB2*, *CACNA2D4,* and *TMEM16B*. Note that although no one gene is PRC specific, their combination is unique for adult PRC. *HCN1* is a member of the Hyperpolarization-activated Cyclic Nucleotide-gated (HCN) family of ion channels [157,158] expressed by all primary sensory neurons [159] and codes for an hyperpolarization-activated current (I_h_), which plays a role in the switch between rods and cones operation upon luminance changes from scotopic to photopic light levels [160]. *KCNB1* is a member of the potassium channel superfamily widely expressed in the nervous system [161] and codes for the pore forming subunit K_V2.1_ which in photoreceptors interacts with the accessory subunit Kv8.2 (coded by *KCNV2*) to generate a heteromeric complex [162] carrying a depolarization-activated current (I_Kx_) [163] that contributes to set PRC membrane potential in darkness. *CACNA1F* is a member of the L-type subfamily of high voltage-activated calcium channels and codes for Cav1.4, the pore forming subunit that partakes with accessory subunits (Ca_Vβ2_ coded by *CACNA2B* and Ca_Vα2δ4_ coded by *CACNA2D4*) in assembling the non-inactivating calcium channel of photoreceptors synapse required to support synapses formation and maintenance [164,165], and the continuous transmitter release in darkness by ribbon synapses of adult PRC (reviewed in [166]). Calcium influx in the synaptic terminal activates a voltage- and calcium-activated current (I_Cl(Ca)_) through channels coded by *TMEM16B*. In mice with defects in the accessory subunit α_2_δ-4 (coded by *Cacna2d4*), the synaptic terminal is disorganized, and I_Cl(Ca)_ is lost [167], indicating it represents a sensitive indicator of PRC synaptic terminal organization and calcium channels assembly.

The progressive acquisition of the electrophysiological fingerprint of PRC in hiPSC-derived ROs is shown by patch-clamp recordings performed at early, middle, and late time in culture, that is, at day (D) 90 (i.e., nearly w13), D150 (nearly w21), and D200 (i.e., nearly w29). In particular, I_h_ could not be recorded at D90, and its amplitude progressively increases at D150 reaching at D200 values similar to those of adult primate rods. Authors also report a non-significant reduction of the outward current, which includes I_Kx_ carried by heteromeric K_V2.1_/K_V8.2_ channels. A decrease in the K_V2.1_/K_V8.2_ ratio has been reported to reduce the outward current K [162], suggesting the developmental changes may reflect an increased expression of *KCNV2* (which codes for KV8.2), driven by the upregulation of the rod-specific transcription factor NRL [168]. Overall, these data suggest PRC in D200 hiPSC-derived ROs acquire the electrophysiological fingerprint of primate photoreceptors, suggesting they are progressing along their developmental trail up to D200.

After 300 days in vitro, i.e., w42–43 or the average gestational time in humans, ROs derived from hiPSC through the embryoid bodies formation develop elongated structures at their periphery. These elongated structures stain positive for rhodopsin, suggesting ROs at w42–43 in culture develop OS-like structures. However, the shape of these OS-like structures appears somewhat different from the morphologies of mature OS of mammalian rods, lacking straight and smooth OS attached to irregularly shaped IS. Although it may be surmised that handling and fixation may have damaged these bona fide OS, electron microscopy did not show evidence for elongated OS with hundredth piled disks, as expected for mature rods, and limited evidence is available about the light-responsiveness of PRC at D300 (i.e., w42–43) [136]. In keeping with the notion of functional channels and transporter localized at axon terminal at D200, it is interesting to note that PRC of hiPSC-derived ROs at 300 days in culture express some components of ribbon synapses and patch-clamp recordings provided clear and compelling evidence by capacitance-tracking of vesicle fusion in response to depolarization-activated calcium entry through non-inactivating calcium channels, indicating functional ribbon synapses in ONL cells of hiPSC-derived ROs.

Because of photoreceptors involvement in many degenerative retinopathies, in the following, we will focus on the obstacles that still hamper photoreceptor functionality in ROs. We will also look for potential solutions by critically reviewing the somewhat fragmented data accumulated during the last 40 years on the SRS microenvironment, where photoreceptors maturation occurs in vivo.

## 3. The SRS Microenvironment

Photoreceptor cells have a peculiar anatomical organization in that their cell bodies lie within the ONL, while IS and OS lie within the subretinal space (SRS) (Figure 1A). Accordingly, the retina can be subdivided into two distinct compartments [169]. The inner compartment, also referred to as the Muller cell compartment, extends between the inner limiting membrane and the OLM. The SRS is a closed extracellular compartment several tenths of microns wide in mice [170] and is the remnant of the original optic vesicle cavity that separates the retina from the RPE. The SRS represents the interface between the inner retinal compartment and the RPE, critical for photoreceptors high oxidative and glycolytic metabolism that supports phototransduction and OS renewal, the latter occurring at an average daily rate of about 10% [170]. RPE functional characteristics are tightly controlled to provide critical support to photoreceptor metabolism and viability. A comparable structure is absent in ROs, and we will summarize data about SRS anatomical and functional features that may prove critical to support IS and OS morphogenesis in iPSC-derived ROs.

### 3.1. SRS Structures

The outer retinal compartment extends between the OLM and Bruch’s membrane and includes the SRS and RPE. The OLM represents the border between the two compartments and appears in histological sections as a demarcation line between the ONL and the SRS [171]. The SRS is a privileged extracellular milieu into which the apical domains of photoreceptors project. RPE and OLM intercellular junction complexes circumscribe the SRS (tight and adherence junctions, TJ and AJ) on their lateral cell surfaces [172], as for all epithelial layers.

The OLM is a continuous sheet of unique AJ connecting the external end-feet of Muller cells with the proximal part of the photoreceptor IS as they emerge from the cell body (heterotypic junction) and occasionally Muller cell to Muller cell and photoreceptor to photoreceptor (homotypic junction) [171,173]. In mice, the OLM is first discernible by PN5 [174].

The AJs in OLM determine apical-basal polarity in photoreceptor cells [175], provide structural support for motile photoreceptors [171], and help them maintain their orientation to incoming light. AJs also provide a semipermeable diffusion barrier, preventing the diffusion of proteins with Stokes radii above 30–36 Angströms [176,177,178,179] out of the extracellular space surrounding the IS and OS. As an example, it is a barrier for retinoid-binding proteins [180].

In the OLM, heterotypic junctions contain proteins from both AJ and TJ. AJs consist of transmembrane protein E-cadherin, intracellular components p120-catenin, β-catenin, and α-catenin [181]. AJs interact with a cytoplasmic plaque comprised of adaptor proteins expressed in TJs as CRB1 and zonula occludens 1 (ZO-1) [173,182,183] and junctional adhesion molecules (JAM) [184]. These specific TJ proteins are detected in the OLM subapical region (SAR) and probably assemble differently in the junctions between Müller cells and rods, Müller cells and cones, and between cones and cones [185]. The mammalian retinal CRB comprises at least one of the three CRB family members, CRB1, CRB2, and CRB3, associated with multiple PDZ proteins 1 (Mupp1) and membrane-associated palmitoylated protein (MPP) [186,187]. The core CRB localized at the SAR [188,189] has a prominent role in controlling apical-basal polarity, acting as a sensor for cell density, and is an essential component of the intracellular scaffold for the assembly of the protein complex at the AJs [189,190,191].

In photoreceptors, the separation of their apical and basal compartments is critical for correct functioning in cell-to-cell adhesion, intercellular signaling, directional transport of molecules, and correct tissue formation. In mouse cone cells, the development and maintenance of IS and OS critically depends on miRNA 182 and 183 [149], which represent over 70% of cone miRNA and are also highly expressed in rods (reviewed in [150]). Intriguingly, these miRNAs also control the expression of *Crb1* [151].

Several labs reported the presence of an OLM in ROs generated from mouse [192,193] and human [53,136,194] iPSC, respectively at D2–25 and D150–190, the time of IS emergence from PRC in the ONL. The presence of the OLM at the time of IS emergence suggests these ROs activate the transcriptional switch preceding IS and OS maturation. However, ROs generated from mouse iPSC and cultured up to D60 never generated OS of length comparable to those in the adult mouse retina [193].

OLM development may represent a required, but not sufficient, step for PRC maturation, and OLM roles may not be limited to its contribution to the barrier between SRS and retina and to provide polarity signals. Muller glial cells digitations extend up to half IS length and affect the precise spatial organization of nascent IS in a honeycomb-like structure [195]. This precise spatial arrangement of IS may be critical for the precise localization of IS elongated mitochondria, which associate to the plasma membrane and position in register with mitochondria of neighboring IS. In regards to the relevance of mitochondria for energy production, the OLM may provide a polarity cue critical for constraining mitochondria positions across IS and optimize energy production in PRC. Importantly, the morphological maturation and the anatomical arrangement of IS mitochondria in the mouse start around PN5-7 and reach completion around PN20 [195], when OS approach their adult length, a finding consistent with OLM role in OS maturation. The distribution and localization of mitochondria in IS of ROs in long-term culture may provide insights into their morphological and functional maturation.

Dysfunctional Crumbs proteins have been associated with different types of retinal degeneration in humans [175]. Mutations in CRB1 cause autosomal recessive LCA, several subtypes of autosomal recessive RP [196,197], and autosomal dominant pigmented paravenous chorioretinal atrophy [198]. Loss of CRB1 disrupts the OLM complexes at the SAR and the AJ [182,199].

In the mouse model of retinal degeneration 8 (rd8), a single base deletion in *Crb1* causes a frameshift and premature stop codon, which truncates the transmembrane and cytoplasmic domain of CRB1. In this model, staining for AJs is discontinuous and fragmented. Shortened photoreceptor IS and OS are observed as early as 2 weeks after birth, suggesting a developmental defect in these structures rather than a degenerative process. Photoreceptor degeneration is observed only within retinal spotting regions, predominantly in the inferior nasal quadrant of the eye, caused by retinal folds and pseudo rosettes [200].

In *Crb1*^–/–^ mice, the retinas are initially normal, but in 3–9 months, they develop localized lesions where the integrity of OLM is lost, and giant half rosettes are formed. These data suggest that CRB1 is not essential for the initial assembly of the AJs between photoreceptors and Muller Glia cells but rather for their maintenance during exposure to light [182].

A recent study reveals that the CRB2a intracellular tail promotes AJs apical distribution in zebrafish retinal and lens epithelia, and the extracellular region of CRB2a drives AJs transformation from punctum adherens into stable zonula adherens [201]. The OLM disruption, either chemically or genetically induced, leads to aberrant localization of retinal cell bodies into the SRS at foci of adhesion loss followed by rosettes formation, a hallmark of loss of polarity in a CRB-dependent manner [191]. In the developing retina of double *Crb1*/*Crb2* KO mice, this leads to an initial increased retinal thickness due to increased proliferation of late retinal progenitors, followed in the mature retina by increased apoptotic cell loss [202].

On the SRS RPE side, junctional complexes separate cell surface membrane into apical and basolateral domains [203] and set the characteristics of the outer blood–retinal barrier (oBRB) regulating metabolites trafficking between the retina and choriocapillaris (ChC) of the choroidal vascular bed [204] via the SRS. RPE TJs are composed of transmembrane proteins, such as Occludin (OCLN); Claudin 19 (CLDN19); MARVEL; and JAM-A,-B,-C and peripheral membrane proteins ZO-1,-2,-3 [204], that work as scaffold proteins binding the transmembrane proteins to each other and the cytoskeleton [205]. Considering TJ relevance for RPE function maintenance, their dysfunction may play a relevant role in a range of ocular pathologies. Disruption of RPE cell–cell junction promotes the production of Vascular Endothelial Growth Factor (VEGF) [206], whose overexpression plays an essential role in the pathogenesis of choroidal neovascularization associated with wet age-related macular degeneration (AMD) [207].

Claudin-19 plays multiple roles in RPE and neurosensory retina development and function [208], and *CLDN19* variants in human iPSC affect retinal neurogenesis and RPE maturation [208]. A *CLDN19* variant causes the renal disease familial hypomagnesemia with hypercalciuria and nephrocalcinosis and ocular involvement (FHHNCOI) [208,209]. Affected patients present with disruption of optic disc development resulting in near blindness and horizontal nystagmus.

Impairment of TJ and accumulation of misfolded proteins drive Epithelial to Mesenchymal transition (EMT) in RPE cells (Reviewed in [210]), which could contribute to different retinopathies, such as proliferative vitreoretinopathy (PVR), AMD, and IRDs (Reviewed in [210]).

### 3.2. SRS Biochemical and Functional Features

The mechanisms underlying OS maturation in rod precursors and OS maintenance in adult rods may provide insights into the critical steps preventing OS full maturation in iPSC-derived ROs. A first relevant point is the identification of the SRS as the dynamic environment where OS growth occurs at a steady pace [170] to balance rhythmic disk shedding [143] with worn-out OS phagocytosis by RPE cells preventing retinal degeneration [211]. A second relevant point is recognizing the high metabolic load imposed on photoreceptors by their OS turnover, which may explain their metabolic reprogramming revealed by aerobic glycolysis.

Warburg initially reported the retina reliance on aerobic glycolysis, which is typical of proliferating neoplastic cells [212]. The puzzling notion of cells resorting to the less efficient glycolytic metabolism despite oxygen availability and an overall high metabolic rate has now been explained by the requirement of biochemical pathways alternative to oxidative phosphorylation (OXPHOS) to provide metabolites required by rapid cell growth [213]. Indeed, aerobic glycolysis may contribute a minor fraction of ATP used by rods [214], but it provides intermediates required for lipid synthesis [214,215] to support the high OS turnover in both rods [170] and cones [216].

**Figure 3:**
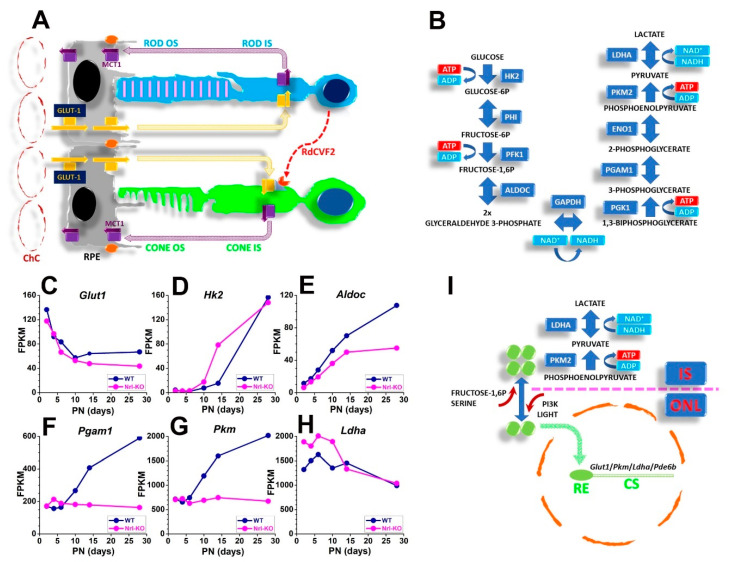
(**A**) Glucose diffuses from fenestrated choriocapillaris (ChC) and crosses the outer Blood Retinal Barrier (oBRB) represented by the tight junctions between RPE cells orange ellipses), reaching the SRS by permeating the basolateral and apical plasma membrane of RPE cells through the facilitated glucose transporter GLUT1 (yellow arrows and boxes). Glucose diffuses along a concentration gradient in the SRS (yellow dotted arrows) to reach GLUT1-expressing rod and cone cells. Lactate generated at the end of the glycolytic pathway is exported from photoreceptors through the monocarboxylate transporter MCT1 (purple arrows and boxes) and diffuses to RPE cells. RPE cells transport lactate and convert it to pyruvate for OXPHOS. Rods promote glucose use by cones by expressing and releasing RdCVF, whose receptor couples to GLUT1. RdCVF expression is strongly reduced in Nrl-KO mice (not shown). (**B**) Schematic representation of the primary enzymatic steps of glucose aerobic glycolytic metabolism, with boxes indicating enzyme isoforms expressed by photoreceptors. (**C**–**H**) Datapoints plot expression data (FPKM) for *Glut1* (**C**), *Hk2* (**D**), *Aldoc* (**E**), *Pgam1* (**F**), *Pkm* (**G**), and *Ldha* (**H**) genes replotted from the RetSeq Database (see text) of transcriptome analysis of flow-sorted mouse rod precursors at different developmental ages, from postnatal (PN) days 2, 4, 6, 10, 14, and 28. Blue and magenta circles plot data for rods isolated from wt and Nrl-KO mice, respectively. Note the prominent upregulation of *Pgam1* and *Pkm* after PN6 in Nrl wt mice. (**I**) Fructose 1,6 diphosphate and serine (upward-pointing red arrow) promote PKM2 (green circles) conversion tetrameric assembly, while light and phosphoinositide 3-phosphate kinase (PI3K) (downward-pointing red arrow) promote the dimeric/monomeric forms. The dimeric/monomeric forms enter the cell nucleus (dotted green arrow) to bind response elements and promote the expression of selected genes, such as *Glut1*, *Pkm*, *Ldha*, and *Pde6b*.

The present understanding of photoreceptors metabolism and OS generation has been stepped forward by novel evidence shedding light on the complex relationships occurring in the SRS between cones, rods, and RPE cells. These cells are now better portrayed as symbionts in a metabolic ecosystem organized to meet rods and cones demanding metabolic needs [215,217,218,219]. As shown in Figure 3A, RPE cells provide the outer Blood Retinal Barrier (oBRB) that controls glucose flux to rods and cones from the large and fenestrated choriocapillaris (*ChC)* of the choroidal circulation, a peculiar vascular bed (reviewed in [220]). Glucose permeates across RPE cells basal and apical membranes via the GLUT1 facilitated transporter to reach the SRS and fuel photoreceptors high oxidative metabolism [218,221] and aerobic glycolysis [219]. The observation that the localized loss of GLUT1 in RPE cells leads to OS shortening, photoreceptor loss, and Muller glia activation [222] indicates the critical role of GLUT1-mediated glucose flux through RPE to support OS assembly and photoreceptors viability. Figure 3A also shows rods and cones export lactate via the mono carboxylate transporter (MCT) to the SRS. Early work indicated that about 80% of glucose consumption by the retina generates lactic acid [217].

RPE cells take up lactate by MCT1 (Figure 3A) and convert it into pyruvate to feed the Krebs’s cycle and generate the ATP required to support their functions [215].

The observation of reduced glucose transport by RPE cells upon reduction of lactate availability [215] supports the notion of the mutualistic relationship between rods and RPE cells metabolism. Lactate generation from pyruvate requires the LDH5 isoform of lactate dehydrogenase, which assembles as a homomeric tetramer of subunits coded by *Ldha*. Photoreceptors express *Ldha*, while inner retinal neurons express *Ldhb* [223,224]. Recent evidence indicates *Ldha* expression by photoreceptors is critical for OS genesis, as electroporation of a short hairpin RNA targeting *Ldha* transcript 3′-UTR resulted in reduced IS and OS length of transfected rods [214]. Moreover, data support the notion of a metabolic ecosystem [215] where retinal and RPE cells engage in a mutualistic relationship providing their partner with energetic substrates. The notion of a metabolic ecosystem involving RPE, rods, and cones fits with the observation of rod-derived cone viability factor (RdCVF) isoforms enhancing glucose transport by cones [225]. RdCVF also contributes to cones viability by protecting them from oxidative stress [226]. Muller glial cells overcome their enzymatic limitations by using metabolites provided by photoreceptors, extending the metabolic ecosystem to retinal glial cells, which provide the OLM that separates the SRS from the retina [227].

The retina generates lactate from glucose despite the concurrent oxidative metabolism providing enough ATP to support the Na/K pump operation required to balance ion influx in darkness and prevent Ca^2+^ build-up [228,229,230]. The conversion of a large fraction of pyruvate into lactate in the presence of oxygen is a hallmark of aerobic glycolysis and requires *Ldha* expression to assemble LDH5. However, the metabolic reprogramming associated with aerobic glycolysis is not limited to the high *Ldha* expression and requires the increased expression of several enzymes involved in glycolysis, as shown in Figure 3B.

Metabolic reprogramming of rods leads to the expression of isoforms characteristic of aerobic glycolysis, such as HK2 [231] and PKM2 [214,232,233], the last one playing a pivotal role in promoting aerobic glycolysis in photoreceptors [214,233]. Rod precursors undergo metabolic reprogramming to aerobic glycolysis during postmitotic specification steps, as shown in Figure 3C–H, which plots publicly available data from the RetSeq Database (https://retseq.nei.nih.gov/, accessed on 15 April 2021) [234]. The increased expression of *Hk2*, *Aldo*, *Pgam1*, and *Pkm* after PN5 is consistent with the development of OS and light responses in rodent rods by PN8 and PN13, respectively [235]. Intriguingly, cells lacking the rod-specific transcription factor Nrl fail to upregulate *Pkm* and *Pgam1* expression after PN5. The increased *Pkm* expression in rods matches data indicating rod expression of the alternative splicing variant *Pkm2* [214,223,227,233], which plays a crucial role in driving the metabolic reprogramming of photoreceptors.

PKM2 may assemble as a homotetramer with high enzymatic activity or as a monomeric/dimeric form with reduced enzymatic activity [236]. Glycolytic intermediate fructose 1,6 diphosphate [237] and serine [238,239] stabilize the homotetramer, promoting glucose metabolism via OXPHOS. On the other hand, tyrosine 105 (Y_105_) phosphorylation reduces PKM2 association as a homotetramer and stabilizes the monomeric/dimeric form [240]. As shown in Figure 3I, PKM2 phosphorylation on Y_105_ promotes its nuclear translocation, where it may bind the response element of genes involved in aerobic glucose metabolism, such as *Glut1*, *Ldha*, and *Pkm* [241,242]. In PRC, PKM2 localizes to IS and cell nuclei in both rodent and primate retina [223]. Suppression of PKM2 levels by a shRNA induces a reduction of OS length [214], consistent with the key role of PKM2-driven metabolic reprogramming for OS renewal. bFGF induces PKM2 phosphorylation on Y_105_ [214], consistent with FGFR1 localization in the ONL of human [243] and rodent [243,244] retinas. Of possible functional relevance for rod function is the observation that light also triggers Y_105_ phosphorylation [233], and dimeric PKM2 upregulates the expression of the rod-specific phosphodiesterase isoform *Pde6b* [245].

### 3.3. Oxygen Levels in the SRS and the Outer Retina

**Figure 4:**
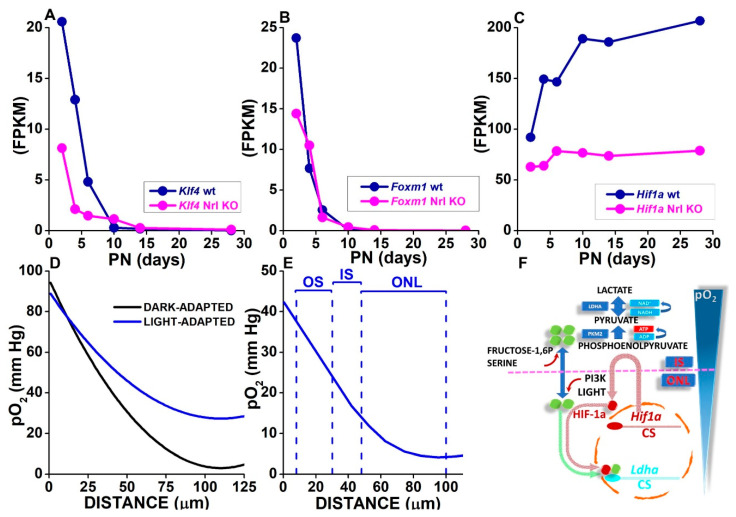
(**A**–**C**) Datapoints plot expression data (FPKM) for *Klf4* (**A**), *Foxm1* (**B**), and *Hif1a* (**C**) genes replotted from the RetSeq Database of transcriptome analysis of flow-sorted mouse rod precursors at different developmental ages, from postnatal (PN) days 2, 4, 6, 10, 14, and 28. Blue and magenta circles plot data for rods isolated from wt and *Nrl*-KO mice, respectively. (**D**) Curves are drawn according to a diffusion-based model using average values from best-fitting curves for pO_2_ changes measured by oxygen-sensing microelectrodes from ChC to ONL in dark-adapted (black) and light-adapted (blue) cat retina (see text). (**E**) The blue curve plots average values from best-fits of pO_2_ values in light-adapted mouse retinas. The upward dashed lines were drawn at positions corresponding to the OS tips, the OS/IS junction, the OLM, and the OPL (left to right), from the CT optoretinogram (see text). (**F**) Scheme of synergic activation of *Ldha* transcription by dimeric/monomeric PKM2 and HIF-1a. The downward blue triangle represents the pO_2_ gradient across the SRS, with lower levels at the ONL than at the IS. *Hif1a* transcription translates into HIF-1a transcription at the IS and may escape degradation by reduced pO_2_ levels, then shuttling back to the cell nucleus to establish a complex with dimeric/monomeric PKM2 and bind the *Ldha* promoter to increase its transcription.

*Ldha* expression represents a critical step for pyruvate conversion to lactate, which controls glucose flux from ChC to the SRS through RPE cells [215] and hence for OS turnover [214]. Transcription factors expressed by rods, such as KLF4, FOXM1, and HIF-1α, may directly control *Ldha* expression in rods (reviewed in [246]). KLF4 represses *Ldha* expression in terminally differentiated epithelial cells, and *Klf4* downregulation correlates with increased *Ldha* expression in pancreatic tumors [247]. FOXM1 plays critical roles during development and mitosis execution and affects glycolysis mainly by upregulating phosphoglycerate kinase (*Pgk1*) and *Ldha* expression. Increased FOXM1 promotes glucose consumption and increases LDH activity and lactate production [248]. Intriguingly, despite opposite effects on *Ldha* transcription, both *Klf4* and *Foxm1* expression vanish by PN 10 during rod development, as shown in Figure 4A,B, plotting data from the RetSeq database [234].

These data suggest neither KLF4 nor FOXM1 affects *Ldha* expression during rod precursors specification steps leading to initial OS assembly, challenging the role of these TF in regulating aerobic glycolysis and OS maturation in rods.

On the other hand, *Hif1a* upregulation during development may affect the metabolic reprogramming of adult rods via cis response elements upstream of *Ldha* transcription start site [249], including a HIF essential binding site [250]. HIF-1 is a heteromeric protein assembled from HIF-1β and HIF-1α subunits and controls cell responses to hypoxia [251]. As shown in Figure 4C, *Hif1a* is already highly expressed at PN2, and its expression further increases after PN5, suggesting it may contribute to *Ldha* upregulation during OS development and in adult rods.

A critical factor in HIF-1 signaling is the posttranslational control over HIF-1α stability by oxygen partial pressure (pO_2_). In normoxia, HIF-1α undergoes degradation by the 26S proteasome upon O_2_-regulated propyl hydroxylation at two key residues [252,253], thus preventing HIF-1 assembly [254]. Accordingly, HIF-1 transcriptional control over *Ldha* requires HIF-1 heterodimer stabilization by low pO_2_ levels in photoreceptor nuclei, i.e., at the ONL levels, a concept hard to reconcile with the high oxidative metabolism of photoreceptors.

Measurements by O_2_-sensitive microelectrodes across retinal layers, SRS, and RPE of the dark-adapted cat retina indicate a steep pO_2_ gradient across the SRS, falling from a value higher than 70 mm Hg close to the ChC to almost 0 mm Hg in a region proposed to correspond to the ONL [218]. The black curve in Figure 4D plots pO_2_ levels computed according to average values generated by fitting a one-dimensional diffusion model to experimental data [218]. The minimum pO_2_ level close to 0 mm Hg measured in the dark-adapted cat retina appears lower than the 35–40 mm Hg reported for the human cortex and closer to values reported for several neoplastic tissues (reviewed by [255]).

As shown by the blue curve in Figure 4D, the minimum pO_2_ increases during light exposure [218], indicating a retinal area, possibly corresponding to the ONL, experiences low O_2_ conditions in darkness and could then be considered hypoxic [255].

Similar pO_2_ profiles across the SRS and retina have been obtained in the light-adapted mouse retina [256] (Figure 4E), and the comparison with retinal layers thickness in vivo provided by the CT optoretinogram [170] indicates the pO_2_ minimum effectively coincides with the ONL and the proximal IS, as initially proposed in the cat retina [218].

A possible model, integrating available molecular data on HIF-1α and PKM2 control over *Ldha* expression with pO_2_ gradient across the SRS, is illustrated in Figure 4F. Low pO_2_ levels in darkness may stabilize HIF-1α and promote *Ldha* expression and lactate production driving the increase in OS length observed in mice kept for days in darkness [214]. This model suggests that in darkness low pO_2_ levels in the ONL may promote aerobic glycolysis via HIF-1α stabilization and will sum up with the lack of OS disk shedding triggered by light onset [170].

Interestingly, HIF-1α also upregulates *Pkm* expression [241,257]. As shown in Figure 4F, dimeric PKM2 (green) may act as a HIF-1α coactivator to promote the transactivation of HIF-1 target genes [241]. This mechanism provides PKM2-specific positive feedback that amplifies the response to HIF-1α and induces the metabolic reprogramming of cells by promoting the transcription of genes involved in aerobic glycolysis, such as *Pkm*, *Glut1*, and *Ldha* [241].

This evidence may indicate that the precise spatial arrangement of the SRS is constrained by the requirements of aerobic glycolysis-based OS generation. Relatively high pO_2_ supports OXPHOS by IS mitochondria, and hypoxic conditions at the nuclear region (ONL) stabilize HIF-1α and promote *Ldha* and *Pkm* expression. The control over *Pkm* and *Ldha* by HIF-1α may represent a critical step for the metabolic reprogramming underlying the operation of the metabolic ecosystem supporting OS development, which requires fine-tuning. In agreement with this notion, *Pkm1* upregulation increases the expression of genes coding for enzymes of the glycolytic pathway in rod photoreceptors of Pkm2^−/−^ mice, and the subsequent loss of aerobic glycolysis protects rods from degenerating in response to long-term retinal detachment [258], i.e., from chronic hypoxia. These findings indicate metabolic reprogramming toward aerobic glycolysis may only operate under tight-controlled pO_2_ and glucose levels. Accordingly, HIF-1α may act as a double-edged sword in photoreceptors, as shown by the slow rod degeneration in mice with about 50% of rods lacking the expression of Vhl factor [259]. *Vhl* codes for a protein that binds proline-hydroxylated HIF-1α and promotes its proteasomal degradation [260], and *Vhl* loss mimics hypoxia-driven HIF-1α increase. Indeed, preventing *Hif1a* expression rescues the slow rods degeneration in *Vhl* mice, pointing to a causal role for increased HIF-1α in reducing rods viability [259]. These data fit with the notion of a critical role of the precise spatial tuning of pO_2_ values to permit OXPHOS at the IS while allowing a long-term moderate HIF-1α stabilization in the nuclei.

Intriguingly, one-week-long detachment of primate retina induced by the injection of a balanced salt solution in the SRS leads to OS loss but spares the IS [261]. This observation suggests that distancing the retina by just 100 µm from the RPE, i.e., leading to hypoxia by increasing the diffusion length through the SRS, severely impairs OS renewal via a selective mechanism that does not affect neither IS nor rods viability. Indeed, retinal reattachment led to a slow recovery of OS length, which took over 30 days to complete [261], with the cone recovery rate exceeding that expected from the 10 days estimated for human cones OS turnover from shedding [216].

It is interesting to note that retinal detachment may not simply affect rods because of reduced O_2_ availability. Increasing O_2_ saturation of breathed air may bring back the outer retinal surface pO_2_ back at its normal levels, but O_2_ consumption fails to recover to its regular rate [262]. It is possible to explore the impact of the main factors affecting the diffusion length of glucose and O_2_ using the one-dimensional diffusion equation that models metabolite supply from a constant-concentration source, such as the ChC. Equation (4) in [263] provides a simple formula for the maximum diffusion length (L_MAX_), i.e., the diffusion distance where the metabolite concentration attains the 0-level from its initial concentration at the source (C_0_):

(1) $$L_{\mathrm{MAX}}=\sqrt{\frac{2C_{0}D}{Q}}$$

where D is the diffusion coefficient, and Q is the metabolite consumption rate. Retinal detachment increases the distance between ChC and IS, causing IS to experience very low pO_2_ and glucose levels. According to Equation (1), increasing pO_2_ in the breathed air may increase C_0_ and L_MAX_ and normalize pO_2_ levels at the IS, but it will likely leave rods and cones with insufficient glucose levels to support OXPHOS [262].

For optimal conditions for OS growth, the SRS microenvironment should match L_MAX_ for O_2_ and glucose. The three parameters in Equation (1) may be pretty similar for glucose and pO_2_ and provide similar L_MAX_ for these metabolites. The ratio between glucose and O_2_ consumption rates (Q) may be inferred considering the conversion into lactate of about 80% glucose consumed by the retina and assuming the remaining glucose is used to fuel OXPHOS. Under this assumption, of every 5 glucose molecules used by the retina, 1 will go to OXPHOS, which requires 6 O_2_ molecules, suggesting similar Q values for glucose and pO_2_. The initial concentration of glucose (C_0_) may differ from its plasmatic value at ChC (about 5 mM), as it needs to cross twice the plasma membrane of RPE cells via the facilitated transporter GLUT1, which requires a concentration gradient across both the basolateral and apical membranes to let glucose cross the oBRB. Considering GLUT1 K_m_ for glucose (1–2 mM), a C_0_ close to the upper value reported for the human brain using microdialysis (median 1.6–1.7 mM with a range 1.15–4.13 mM, [264,265]) is reasonable.

Considering the similar consumption rate for O_2_ and glucose (see above) and assuming the highest value of the range (4 mM) as the standard C_0_ value for glucose diffusion after the RPE, a concentration change within the SRS similar to that measured for pO_2_ would end up with glucose concentrations at the IS close or even lower than GLUT1 Km for glucose. The functional impact would be a transport rate substantially lower than the maximum transport velocity, possibly limiting glucose use by photoreceptors compared to O_2_.

A factor that may shape glucose and pO_2_ concentrations drop along the SRS is the diffusion coefficient (D), whose value according to the Stokes–Einstein relation depends on medium viscosity, as shown in Equation (2):

(2) $$D=\frac{\mathrm{kT}}{6{\pi \eta r}_{s}}$$

where k is the Boltzmann constant, T is the temperature in K°, η is the viscosity, and r_s_ the Stokes’ radius of the molecule. Interestingly, the extracellular matrix may affect the viscosity of the SRS microenvironment, but it also has effects on D that depends on matrix characteristics, such as its mesh size. As reported for a silated-hydroxypropylmethylcellulose (Si-HPMC) hydrogel, the O_2_ diffusion coefficient dropped from 2.8 × 10^−9^ m^2^ s^−1^ in culture medium to less than 3 × 10^−10^ m^2^ s^−1^ in a 2% hydrogel, i.e., a nearly 10-fold reduction [266]. Intriguingly, the time required to reach 50% of the equilibrium concentration increased with hydrogel concentration much more for O_2_ than for glucose [266], indicating hydrogel characteristics may modulate the diffusion coefficient of solutes to tune their L_MAX_ to optimal values for a given 3D environment. Interestingly, similar effects have been found for hydrogels of the extracellular matrix of biological tissue (see Table 1 in [266]).

These findings suggest hydrogels of the extracellular matrix may influence cell fate and differentiation and modulate the availability of crucial nutrients, such as glucose and O_2_, to support cell growth and differentiation by fine-tuning their L_MAX_ parameters via control over their diffusion coefficients. The influence of the extracellular matrix over nutrients availability may add up on other effects on ROs development [267].

## 4. Bioengineering the SRS Microenvironment

According to the evidence discussed in Section 3.2 and Section 3.3, oxygen and glucose spatial gradients within the SRS microenvironment are critical for OS turnover, the viability of metabolically reprogrammed photoreceptors, and the development of mutualistic relationships with RPE cells. It is generally surmised that static culture systems may not provide enough O_2_ and glucose to support OS turnover in retinal photoreceptors, but the point has never been addressed. It is relevant to note that, contrary to the effects of pO_2_ on the maturation of postmitotic rod precursors presented and discussed in Section 3.2 and Section 3.3, several protocols included culturing embryoid bodies or ROs in the presence of either increased (40% rather than 20%) [127] or time-limited decreased (5% for 10 days followed by 20%) O_2_ levels [268]. Additional variants for hESC-derived ROs used intermittent exposure to 40% O_2_ levels [269] at relatively early developmental stages (D38, D50, and D62). However, in these studies, low pO_2_ levels were used at specific time points of early ROs development, to affect progenitor cells proliferation and increase the number of photoreceptor-fated cells [268]. A second related issue is whether the dynamic culturing conditions provided by available bio incubators may reproduce the SRS microenvironment to support metabolic reprogramming of photoreceptors and OS maturation. Assessing whether either static or dynamic culture conditions may secure enough glucose and O_2_ to support OS growth and photoreceptors operation in a native retina bears a clear relevance to the issue of whether or not 3D ROs may develop full-length OS, similar to native retinas.

### 4.1. Limitations of Conventional Static Culture Systems

Retinal glucose consumption has been measured in primates and non-primate mammalian species. For the rat retina, the rate of glucose consumption has been estimated at 1.6 µmoles/retina/h [217]. Assuming the dry weight of the mouse retina is about 1/10 of the rat retina, under the hypothesis that rat and mouse retinas have similar metabolic rates per unit of dry weight, the glucose metabolic rate of the mouse retina should be 0.16 µmoles/retina/h. Considering a dish containing 2 mL medium with 4.5 mM glucose (about 0.08 g/dL), the amount of glucose available for metabolism before its concentration drops to 1 mM is 7 µmoles. Assuming glucose utilization proceeds unabated at the rate of 0.16 µmoles/retina/h, it would take about 44 h to reach a glucose concentration of 1 mM (0.018 g/dL), corresponding to a plasma glucose level associated with brain damage [270]. According to these estimates, static culture conditions with media change every 2/3 days may bring the retina close to severe hypoglycemic conditions.

The glucose consumption rate has been measured with oxygen supporting light responsiveness through OXPHOS [217]. Light-induced 11-cis retinal isomerization triggers a metabolic cascade leading to the closure of cGMP-sensitive channels (CNGs) open in darkness [271]. CNGs have a high permeability to both Na^+^ and Ca^2+^ ions [272] (for review, see [273]), causing a steady influx of cations in darkness [228], balanced by Na/K ATPase and Na:Ca;K exchanger [274]. In both primate and rodents rods, the block of the Na/K ATPase leads to a rapid and irreversible loss of the dark current [228], indicating a high turnover for Na^+^ in mammalian rods at body temperature. Furthermore, the activation of a hyperpolarization-activated current in primate [275] and non-primate [276,277,278] rods generates a Na^+^ influx through hyperpolarization-activated channels requiring Na/K ATPase operation to prevent Na^+^ build-up and calcium increase [229]. The O_2_ requirement of 7 mL min^−1^ for 100 g tissue estimated for the dark-adapted cat outer retina [218] could be scaled down to the mouse retina and converted to an O_2_ consumption rate (Q) of 47 µMoles O_2_ s^−1^ L^−1^ in darkness and 20 µMoles O_2_ s^−1^ L^−1^ in bright light. Table 3 reports L_MAX_ values computed inserting Q values either in darkness or in bright light in Equation (1) and the height of culture medium for a 3 cm diameter dish and a 1.1 cm diameter well.

Comparing the computed L_MAX_ with the height of 2 mL medium in a 3 cm diameter Petri dish (2830 µm) or 1 mL in a well of a 12-well plate (2610 µm), it is evident that the height of medium exceeds the computed L_MAX_ by over an order of magnitude, both in light or darkness.

From this analysis, it is clear that an adult mouse retina with fully developed OS may not thrive using conventional static culture methods, failing to receive enough O_2_ to support OXPHOS. It is relevant to note that upon OXPHOS block by KCN, the rat retina increases its already high lactate production by over 50% [217], i.e., glucose consumption increases by over 50%, suggesting that in static culture conditions glucose may take approximately 30 h to approach the critical 1 mM level. In addition, when cultured in a well with 1 mL medium, glucose may fall below 1 mM in less than 15 h.

The same approach could be used to assess the upper limit for the metabolic rate of a 3D RO. The computation indicates that upon reaching a metabolic rate of 1/100 of a dark-adapted mouse retina, L_MAX_ approaches 2730 µm, i.e., a value in between the medium heights in a 3 cm dish and 1.1 cm diameter well, suggesting static culture conditions limit the growth and maturation of iPSC-derived ROs.

### 4.2. Dynamic Culture Systems

Both hamster [280] and mouse [281] retinas cultured at 27 °C in a flow-through apparatus maintain rhythmic melatonin production up to 5 days, providing evidence for improved retinal viability and function using dynamic culturing conditions compared to static culture systems. Intriguingly, light stimulation reverses the melatonin rhythm, indicating retinas remain light-responsive for several days when cultured at 27 °C. The evidence of preserved viability and function up to 5 days, when cultured with continuous perfusion, matches ONL thickness and IS length preservation. However, after 6 days in culture, OS appear swollen and shorter than freshly isolated retinas, suggesting dark current reduction at 27 °C [282] allows photoreceptors survival in low pO_2_ levels by relieving their dependence on OXPHOS. Still, the reduced metabolic rate may impair the synthesis of new building blocks required for OS turnover [214,215,233,245]. According to Section 3.3, the flow-through apparatus may prevent the glucose shortage expected from static culture conditions, but O_2_ availability may fall short of supporting OXPHOS at 37 °C, requiring a lower temperature to match O_2_ request by OXPHOS with O_2_ diffusion rate.

An increase in flow rate may overcome the limited O_2_ diffusion by mass transfer and provide enough O_2_ to support OXPHOS, but it may cause tissue damage by shear stress, a shortcoming of the flow-through apparatus. Shear stress is also an issue with stirred bioreactors, which exploit mass transfer to overcome the O_2_ diffusion limits. Stirred bioreactors have been used for the development of iPSC-derived cerebral organoids [283] and ROs [284]. Cerebral organoids may fail to increase in size due to limited O_2_ diffusion within the tissue [283]. This problem may be solved by culturing an organoid slice at the air–liquid interface [285], a condition supporting axonal growth and the assembly of neuronal networks for up to one year. In human iPSC-derived ROs, culturing in a stirring bioreactor led to increased ONL thickness associated with increased progenitor proliferation. Culturing in a stirred bioreactor also decreased apoptosis of postmitotic cells and led to the earlier development of IS and OS compared to static culture conditions [284]. However, at late developmental times, organoids miss OS upon prolonged culturing in a stirred bioreactor compared to static cultures [284]. Although the reasons underlying OS loss with time in the stirring bioreactor had not been identified, fully developed OS are a lipid-rich compartment whose density is lower than the remaining organoid and, as such, may increase the 3D structure tendency to float and move in medium, similar to retinal slices ticker than 150 µm (GCD personal observations). 3D ROs of increased OS length may relocate closer to the stirring vanes and experience increasing shear stress, ultimately breaking the mechanically sensitive cilium connecting IS and OS. However, no compelling experimental support for this hypothesis is available yet.

The National Aeronautics and Space Administration (NASA) initially developed the Rotating Wall Vessel (RWV) to investigate microgravity impact on cells and tissue. However, the RWV is also a culture system able to minimize the issue of unstirred layers typical of static culture systems while minimizing shear stress, therefore apt for the development of 3D tissues [286]. A clear description of the fundamental physical principles underlying the RWV bioreactor operation and its main trade-offs has already been presented [287].

The multipotential human retinal progenitor cell line (KGLDMSM) [288,289,290] grown attached to laminin-coated microcarrier beads in the RWV developed progressively larger aggregates, up to a size of several hundred microns, by bead fusion [291]. Tissue growing between fused beads generated 3D-like structures, with several cells acquiring a morphology reminiscent of photoreceptors, with IS and OS-like structures [291], despite these structures lack evidence of their organization in laminated structures as ESC- or iPSC-derived ROs. Intriguingly, the same cells grown in a stirring bio-incubator developed large cell aggregates similar to those grown in the RWV but failed to evolve differentiated morphologies reminiscent of IS and OS [291].

The use of RWV also improved the development of murine ESC-derived ROs compared to static culture [192]. In general, RWV incubation led to accelerated development of iPSC-derived RO compared to static cultures, which required more time to reach the same developmental stage of RWV-cultured ROs [192]. This notion has also been substantiated by transcriptome analysis, which indicates that murine ESC-derived ROs reach a maturation stage corresponding to a PN6 mouse retina but fail to show further progress, independently of the culturing conditions [192]. Considering that RWV provides ROs with improved nutrient availability, the most straightforward interpretation is that ROs require additional factors not available in vitro, unrelated to O_2_ and glucose supply, to promote transcriptional changes leading to enhanced expression of genes involved in OS biosynthesis and phototransduction cascade components. A difference between the retinal progenitor cell line [291] and ESC-derived ROs [192] cultured in RWV is the coculturing of the progenitor cells line with the D407 human RPE cell line [292] in a 1/3 ratio, mimicking the ratio occurring in the eye between RPE cells and photoreceptors. Note that although transcriptomic data indicate the evidence of a gene cluster signaling the occurrence of RPE cells in ESC-derived ROs [192], there is no indication of the ratio between RPE cells and photoreceptors. However, considering the lack of molecular characterization of photoreceptor-like cells [291], the role of RPE may not represent the only difference between the two models, and RPE role in driving photoreceptor OS maturation requires a detailed investigation.

A hint on the functional role of RPE cells in OS development in culture comes from a system merging organ-on-a-chip bioengineering with iPSC-derived ROs. The retina-on-a-chip (RoC) platform aims at reproducing in vitro the micro-physiological environment where IS and OS mature, usually missing in conventional culture systems, either static or dynamic. The organ-on-a-chip retina features a two-chamber system, assembling a human iPSC-derived ROs in close contact with an RPE cell layer in the upper compartment, with O_2_ and nutrients diffusing from the lower compartment perfused by culture medium, mimicking ChC role in the eye [21]. This design provides a constant supply of nutrients and O_2_, overcoming a critical limitation of static culture systems. Compared to stirred bioreactors, the RoC provides O_2_ and nutrients supply by diffusion rather than mass transfer, thus avoiding tissue exposure to shear stress. The system also offers an advantage over the RWV by avoiding organoids spiraling in the medium with cells moving at a variable distance from the oxygenator membrane that provides O_2_ by diffusion, thereby avoiding exposure of cells to varying pO_2_.

Last but not least, continuous perfusion of the RoC avoids the periodic interruptions of incubation, required for medium replacement by both stirred bioreactors and RWV. In addition, microfluidic channels in the RoC require far smaller fluid volumes than stirred bioreactors and RWV, a critical issue when using expensive chemical factors for steering iPSC toward a specific fate. RPE cells and ROs were derived in static cultures from human iPSC using specific mixed 2D/3D differentiation protocols [132], confirming the previously reported [132] generation of short IS and OS at 190D, roughly corresponding to the developmental stage of photoreceptors in a human fetus at the beginning of the 3rd trimester of pregnancy. Upon coculturing RPE cells with ROs embedded in the hyaluronan matrix, OS comes close to RPE cells, and RPE cells’ phagocytic activity supports disk shedding [21] and possibly OS renewal in these culture conditions.

The protocol adopted for ROs generation supports OS functionality, as shown by the response to light stimuli in about 10% of rods [132] (but see Section 2.3). The ability of ROs to generate light responses in GC/AC cells has also been reported [136,144]. The finding of the interaction between rods and RPE in the RoC [21], with rhodopsin-positive membrane inclusion suggestive of OS renewal and phagocytosis by RPE cells, represents a significant advance in the generation of functionally mature photoreceptors from human iPSC and the SRS microenvironment for modeling and treatment of IRD.

Further development of the RoC should evaluate the long-term stability of ROs and OS development in the RoC. This evaluation may require critical analysis to assess O_2_ consumption by mature RO and match it with its delivery by diffusion from the media lacking an O_2_ carrier. Estimates of O_2_ consumption by an adult mouse retina provided in Section 3.3 (of 47 µMoles O_2_ s^−1^ L^−1^ for a 10 µL retina) translates into 470 pMoles O_2_ s^−1^ retina^−1^. The 20 µL h^−1^ medium perfusion rate of the RoC provides an amount of dissolved O_2_ of 1.14 pMoles O_2_ s^−1^, clearly lower than the 470 pMoles O_2_ s^−1^ required by an adult mouse retina. Although the mouse retina volume exceeds that of a RO, and retinal full-length OS outnumbers those in the RO, the retinal O_2_ consumption should be regarded as an upper limit estimate for the O_2_ requirement by a RO with fully developed OS.

### 4.3. Biotechnologies and Human ROs-Derived Photoreceptor Precursors in Transplantation

The number of donor photoreceptor precursors transplanted into the SRS may represent an important variable in the outcome of regenerative approaches to cure IRD [104]. It is presently unclear how the number of grafted cells (an extrinsic factor) may affect cone OS maturation, but the finding may lead to a significant paradigm change in transplantation work, which has so far mostly focused on the identification of improved sorting criteria to select optimal engrafting donors and discard inappropriate cells. The lack of substantial improvements in the yield of engrafting donor cells despite their selection by progressively more stringent sorting criteria [101,102,103] may indicate a role for maturation independent factors in transplantation outcome, i.e., for extrinsic in addition to intrinsic factors and their interplay, as detailed in Section 3.2 and Section 3.3.

Data discussed in Section 2 and Section 3 indicate several SRS characteristics, such as glucose and O_2_ gradients, as wells as the presence of a hyaluronan-based extracellular matrix, may also affect transplantation outcomes. Specifically, hyaluronan-based hydrogels may swell in response to the saline volume used to transfer donor cells in the SRS [293]. RPE cells will then transfer H_2_O from the SRS to the perivascular space of ChC (for review see [294,295]), and the water flux may drag transplanted cells toward the RPE. As discussed in Section 3.1 and Section 3.2, the high pO_2_ on the RPE side of the SRS may adversely affect donor cells metabolic reprogramming required to support OS synthesis and turnover.

Ideally, the optimal strategy to improve transplantation yield would take care of three aspects: (a) increase the number of photoreceptors precursors transplanted to the SRS and maximize the seeding density to enhance cell–cell contact; (b) minimize the volume of saline volume transplanted to the SRS to prevent swelling of the hyaluronan-based ECM; (c) keep grafted precursors closer to the retina than to the RPE, to promote the metabolic reprogramming required for IS and OS generation. However, these conditions may not be easily achievable. Increasing the mass of grafted cells within volumes smaller than microliters may increase the adhesion of precursor cells to plastic and needles, causing uncertainties in the number of delivered cells. Even more difficult would be controlling the position of delivered cells within a SRS a few tenths of microns wide.

Micro scaffolds may meet the requirements of high cells per unit volume, minimal volume of transferred water, and the distancing of grafted cells from the RPE. The fabrication of 3D micrometer-size scaffolds has recently been found suitable for the maturation and polarization of photoreceptor precursors isolated from hiPSC-derived ROs [296]. The scaffold has been designed as an array of cup-shaped wells that funnel the cell toward a channel for promoting cells attachment to the cup wall and generate polarization cues for basal neurites extension. This “wine glass” model was originally designed as a reproducible in vitro platform for scalable monolayer model of photoreceptor diseases. This 3D microstructured scaffold design has been recently upgraded by improving the microfabrication technology to reduce wall thickness and increase the number of cells loaded for each well and better mimic the multilayer ONL structure with densely packed cells [297]. This “ice cube” model may better approximate the 3D ONL structure with higher cell numbers for unit volume than the “wine glass” model design and may represent a model for cell replacement approaches meeting the constrain of high seeding density, minimal saline volume, and improved control over cell positioning in the SRS outlined above.

## 5. Conclusions

The introduction of the iPSC-derived ROs technology opened new perspectives for developing cell-based approaches to treat or cure IRD. It is now possible to generate in vitro and sort human donor cells of the appropriate stage for transplantation [81,82,83], corresponding to the end of the second trimester of pregnancy, solving the issues with donor cells otherwise not available for ethical reasons. Furthermore, reports from independent laboratories on human iPSC-derived 3D ROs with rudimentary IS and OS [21,132,284] demonstrate the possibility of generating and isolating properly developed donor cells for transplantation.

These impressive achievements represent an undeniable success that paves the way toward developing a cure for IRD by replacing missing cells with iPSC-derived cells. However, the development of human disease models for discovering novel drug targets and the development of drug tests remains a largely unmet need for late-onset IRD. In vitro modeling of IRD caused by defective genes coding for phototransduction components requires the well-developed OS to investigate the pathogenetic mechanisms associated with the misfolding of highly expressed proteins, such as rhodopsin, or the metabolic overload caused by variants affecting cGMP turnover. Although the functional analysis of hiPSC-ROs indicates their ability to respond to light in parallel with the development of rudimentary OS [82], the critical review of these data in Section 2.3 reveals their responses to light stimuli do not match those of adult human rods [156]. Light responses were reported in rods with the larger holding currents in darkness, suggestive of endogenous cGMP generation, but light does not close most of this large inward current in darkness [82]. These findings could be compared to mouse ciliary margin-derived cells, which generate endogenous cGMP, express cGMP-gated channels, and respond to 8-Br-cGMP by generating an inward current through channels with the biophysical properties of cGMP-gated channels [298]. In these rod-like cells lacking an OS, the current evoked by 8-Br-cGMP current does not correlate with the holding current at −40 mV (i.e., the rod voltage in darkness) [298], suggesting that care should be taken in correlating the holding current at −40 mV with the dark current through cGMP-gated channels.

Patch-clamp analysis shows ONL cells in hiPSC-derived ROs held at −40 mV respond to 250 µM cGMP (a concentration opening nearly 100% of cGMP-gated channels) by generating an inward current of about 100 pA [299]. However, the physiological cGMP concentration in dark-adapted rods is much lower than 250 µM and activates about 1–4% of total channels [300,301], which translates in response amplitudes to saturating light intensities within a range of 1–4 pA. This value is much lower than the light response amplitudes of human [156] and primate rods [275], but consistent with the authors reporting occasionally small light response from ONL cells in their late-stage hiPSC-derived ROs with rudimentary OS [299]. Dark current amplitudes impact on IRD because of the Ca^2+^ permeability of cGMP-gated channels, which impose a metabolic toll on photoreceptors due to the cost of Ca^2+^ extrusion required to balance the steady Ca^2+^ influx in darkness [228,229]. Immature ONL cells in hiPSC-derived ROs with rudimentary OS and reduced dark current amplitudes may not fully recapitulate the Ca^2+^ overload occurring in late-onset retinal degenerations linked to phosphodiesterase defects [112,113,114] and as a consequence may provide insights on disease progression in patients biased on the cGMP increase component independent from the increased Ca^2+^ influx.

Progress toward improved OS generation by human iPSC-derived 3D RO requires an understanding of the roles played in OS maturation by intrinsic and extrinsic factors and their interplay, as shown by PKM2 isoform role in both metabolism and gene expression [214,232,233,245], which appears critical for aerobic glycolysis and OS generation [214,215,222,223,224]. While the recent focus on the role of glucose metabolism in photoreceptor precursors maturation represents a critical conceptual innovation compared to the notion of purely intrinsic mechanism in fate assignment in retinal development [302,303], the possible role of O_2_ in steering photoreceptors maturation remains to be investigated.

3D RO incubation in 5% O_2_ increases retinal progenitors proliferation [192,268], but the lack of awareness on the possible role played by O_2_ in photoreceptor precursors maturation is surprising, considering the well-known roles of O_2_ on gene transcription in other systems [251,304]. In this regard, if a glycolytic intermediate may have pleiotropic effects on gene expression in photoreceptors by promoting the PKM2 tetrameric assembly [236,237,239], O_2_ may affect rods beyond OXPHOS. We put HIF-1α in the context of OS maturation by reviewing data on the sharp pO_2_ gradient in the SRS [218,221,256], the microenvironment where OS and IS develop and mature in the eye. We also stressed the possible connection between PKM2, a master regulator of aerobic glycolysis, and HIF-1α, a critical factor in O_2_-mediated transcriptional regulation, to control *Ldha* expression [241,242].

We believe the critical review of data from different fields and methodologies may help investigators understand the physical constraints required by photoreceptors to thrive in the face of the high metabolic load imposed by the rhythmic renewal of their OS.

According to O_2_ and glucose constraints associated with diffusion within the SRS, the limited O_2_ availability in static culture may impair photoreceptors maturation via the metabolic reprogramming that supports OS turnover, a critical issue in the generation of iPSC-derived ROs. Data on dynamic culture systems indicate an improved functionality for the RoC design [21], which appears to overcome the shear stress associated with mass-transfer of O_2_ and glucose in the case of stirred bioreactors [284] and the diffusion-limited O_2_ supply in RWV [192]. The small volume of incubation medium required by the RoC compared to stirred bioreactors and RWV represents a feature of interest for drug development.

The RoC design may offer the exciting opportunity to investigate the mechanisms involved in material transfer between donor and recipient human photoreceptors in a more accessible and controlled system than in vivo and the factors either promoting or preventing the engraftment of human donor rod precursors in the host human ROs.

To this end, some adjustments would be required to improve photoreceptors maturation and stability over a few weeks by improving O_2_ and glucose availability to iPSC-derived RO cultured as RoC. Improving the RoC operating conditions may prompt the use of hiPSC-derived 3D ROs for IRD modeling and treatment development. In this regard, the optimization of the extracellular matrix may provide a significant improvement toward the optimization of ROs generation [267].

An emerging question is whether the SRS represents a permissive place for donor cell transplantation, a question tightly connected to the low yield of cell engraftment upon transplantation to the SRS. Upon transplantation, rod precursors nuclei may experience a higher pO_2_ level than in the ONL, possibly undermining the cooperative transcriptional control by PKM2 and HIF-1α over genes coding for aerobic glycolysis key enzymes. It is interesting to note that most transplantation studies just mention that donor cells survive in the SRS but provide few insights on their characteristics [63,72,73,74,75,76,77,78,81,95,101,102,117,118,120,145]. In cone precursors transplantation, the SRS donor cell mass never develops an OS despite cells remaining viable for months and staining positive for cone-specific arrestin isoform. The failure of viable cone precursors in the SRS to generate an OS may result from the loss of polarity and/or dissociation and sorting-induced changes. However, data from 3D retina-like structures (i.e., maintaining cell–cell contact and polarity signals) generated within the SRS by forcing the expression in RPE cells of TF promoting neural retina fate suggest dissociation and sorting-associated changes may not explain ROs failure to mature full length OS. Indeed, ROs generated upon forcing Neurogenin-1 and Neurogenin-3 expression in RPE cells under the control of either RPE-specific TF bestrophin or RPE65 [305], respectively, fail to mature IS and OS. Intriguingly, these latter 3D retina-like structures have an opposite orientation than normal retinal tissue, i.e., the presumptive GC cells are closer to the RPE than the presumptive ONL [305], thus preventing the IS of the ectopic retina-like tissue from obtaining enough O_2_ and glucose for supporting OS operation and growth, respectively.

The development of bioengineered RoC assembling iPS-derived 3D ROs with RPE cells may prove suitable to address pO_2_ contribution to OS generation and devise solutions to improve cell transplantation yield.

## Figures and Tables

**Table 1:** Genetic loci and number of genes, with their inheritance mode, for disease categories Retinitis Pigmentosa (RP), Leber Congenital Amaurosis (LCA), Cone or Cone-Rod Dystrophy (CORD), and Macular Degeneration (MD) from the RetNet database (https://sph.uth.edu/RETNET/sum-dis.htm, accessed on 15 February 2021).

Diseases Category	Inheritance Mode	Loci	Genes
retinitis pigmentosa	dominant	23	22
retinitis pigmentosa	recessive	43	41
retinitis pigmentosa	x-linked	5	2
	subtotal	71	65
leber congenital amaurosis	dominant	1	1
leber congenital amaurosis	recessive	13	13
	subtotal	14	14
cone or cone-rod dystrophy	dominant	9	5
cone or cone-rod dystrophy	recessive	17	16
cone or cone-rod dystrophy	x-linked	1	0
	subtotal	27	21
macular degeneration	dominant	14	10
macular degeneration	recessive	4	4
	subtotal	18	14
overall total		130	114

**Table 2:** Genes involved in photoreceptors development and function.

Acronym	Full Name
ABCA4	ATP Binding Cassette Subfamily A Member 4
AIF	Apoptosis-Inducing Factor
ALDOC	Aldolase Isoform C
ARL2BP	ADP Ribosylation Factor Like GTPase 2 Binding Protein
ARL6	ADP-Ribosylation Factor-Like GTPase 6
BMP	Bone Morphogenetic Protein
C-KIT	KIT Proto-Oncogene, Receptor Tyrosine Kinase (CD117)
C2ORF71	Photoreceptor Cilium Actin Regulator
CACNA1F	Calcium Channel A Subunit Subfamily Xx Isoform 1F (Cav1.4)
CACNA2D4	Calcium Channel A2d Subunit Isoform 4 (Cava2d4)
CACNAB2	Calcium Channel Β Subunit Isoform 2 (Cav2.2)
CD24	Cluster of Differentiation 24
CD44	Cluster of Differentiation 44
CD73	Cluster of Differentiation 73
CEP290	Centrosomal Protein 290
CLDN19	Claudin 19
CNGA1	A Subunit Of The Rod Cyclic-Nucleotide-Gated-Channel
CNGB1	Β Subunit of The Rod Cyclic-Nucleotide-Gated-Channel
CRB1	Crumbs Homolog 1
CX3CL1	C-X3-C Motif Chemokine Receptor 1 Ligand
CX3CR1	C-X3-C Motif Chemokine Receptor 1
FGF1	Fibroblast Growth Factor 1
FGF9	Fibroblast Growth Factor 9
FOXM1	Forkhead Box M1
GLUT1	Glucose Transporter Isoform 1
GNAT1	G Protein Subunit A Transducin
HCN1	Hyperpolarization-Activated Cyclic Nucleotide-Gated Isoform 1
HIF-1alpha	Hypoxia Inducible-Factor Subunit Alpha Isoform 1
HIF-1beta	Hypoxia Inducible-Factor Subunit Beta Isoform 1
HK2	Hexokinase Isoform 2
IGF1	Insuline-like Growth Factor 1
IMPG1	Interphotoreceptor Matrix Proteoglycan 1
JAM	Junctional Adhesion Molecules
KCNB1	Potassium Channel Subfamily B Isoform 1 (Kv2.1)
KCNV2	Potassium Channel Subfamily V Isoform 2 (Kv8.2)
KLF4	Kruppel-Like Factor 4
LDHA	Lactic Dehydrogenase A
MCT1	Mono Carboxylate Transporter Isoform 1
MITF	Melanocyte Inducing Transcription Factor
Mupp1	Multiple PDZ Proteins 1
NEUROG1	Neurongenin1
NEUROG2	Neurogenin 2
NRL	Neural Retina Leucine Zipper
OCLN	Occludin
PAX6D	Paired Box 6 Isoform D
PDE6A	Phospodiesterase 6A
PDE6B	Phospodiesterase 6B
PDE6G	Phospodiesterase 6G
PGAM1	Phosphoglyceraldehyde Mutase Isoform 1
Pgk1	Phosphoglycerate Kinase 1
PKM	Pyruvate Kinase
RdCVF	Rod-Derived Cone Viability Factor
REEP6	Receptor Expression Enhancing Protein 6
RETGC1	Retinal Guanylate Cyclase 1
RHO	Rhodopsin
RP1	Oxygen- Regulated Prein-1 (ORP1)
RP2	Retinitis Pigmentosa 2
RPE65	Retinoid Isomerohydrolase 65
RPGR	Retinitis Pigmentosa GTPase Regulator
RS1	Retinoschisin 1
SAG	S-Antigen (Arrestin)
SSEA4	Stage-Specific Embryonic Antigen-4
TMEM16B	Transmembrane Channel Family 16 Isoform B
TTC8	Tetratricopeptide Repeat Domain-Containing Protein 8
VEGF	Vascular Endothelial Growth Factor
VHL	Von Hippel Lindau
VSX2	Visual System Homeobox 2
WNT	Wingless and Int1
ZO1	Zonula Occludens 1

**Table 3:** O_2_ diffusion coefficient (D) is assumed similar in medium and water (2.8 × 10^−9^ m^2^ s^−1^). C_0_, the air/medium interface at 37 °C (2.09 × 10^−4^ Moles L^−1^), converted in Moles L^−1^ from tabulated values in mg/mL (http://www.fao.org/3/ac183e/ac183e04.htm, accessed on 15 February 2021) at 37 °C. Q for the mouse retina was computed assuming a retinal volume of 10 µL and a retinal weight of 9 mg to account for the high lipid content of the OS. The retinal weight is about three times the values reported in Figure S3 of Cerani et al., 2013 [279], but consumption for unit retinal volume is similar.

Medium Volume (ml)	D (Diffusion Coefficient)	C_0_ (pO_2_ in Air × Solubility Coefficient)	Q (O_2_ Consumption Rate × Retina Volume)	L_MAX_ (µm)	Medium Height (mm)
2 (Dark)	2.8 × 10^−9^ m^2^ s^−1^	2.09 × 10^−4^ Moles L^−1^	47 µMoles O_2_ s^−1^ L^−1^	158	2830
2 (Light)	2.8 × 10^−9^ m^2^ s^−1^	2.09 × 10^−4^ Moles L^−1^	20 µMoles O_2_ s^−1^ L^−1^	241	2830
1 (Dark)	2.8 × 10^−9^ m^2^ s^−1^	2.09 × 10^−4^ Moles L^−1^	47 µMoles O_2_ s^−1^ L^−1^	158	2610
1 (Light)	2.8 × 10^−9^ m^2^ s^−1^	2.09 × 10^−4^ Moles L^−1^	20 µMoles O_2_ s^−1^ L^−1^	241	2610

## Data Availability

Table 1 and Table 3, and Figure 3 and Figure 4 have been generated using data publicly available and the database used and their data of access have been referred to in their caption and in text.
